# Supplementary material for: PSPC1-interchanged interactions with PTK6 and β-catenin synergize oncogenic subcellular translocations and tumor progression
Source: Nat Commun. 2019 Dec 16;10:5716. doi: 10.1038/s41467-019-13665-6 (PMC6914800; doi:10.1038/s41467-019-13665-6)
Supplement: Supplementary file 1 — Supplementary Information [file 41467_2019_13665_MOESM1_ESM.pdf]

Supplementary Information

Manuscript:

PSPC1-interchanged interactions with PTK6 and β-catenin synergize oncogenic subcellular translocations and tumor progression

Lang et al.,

Contents:

Supplementary Figures 1-8

Supplementary Tables 1-5

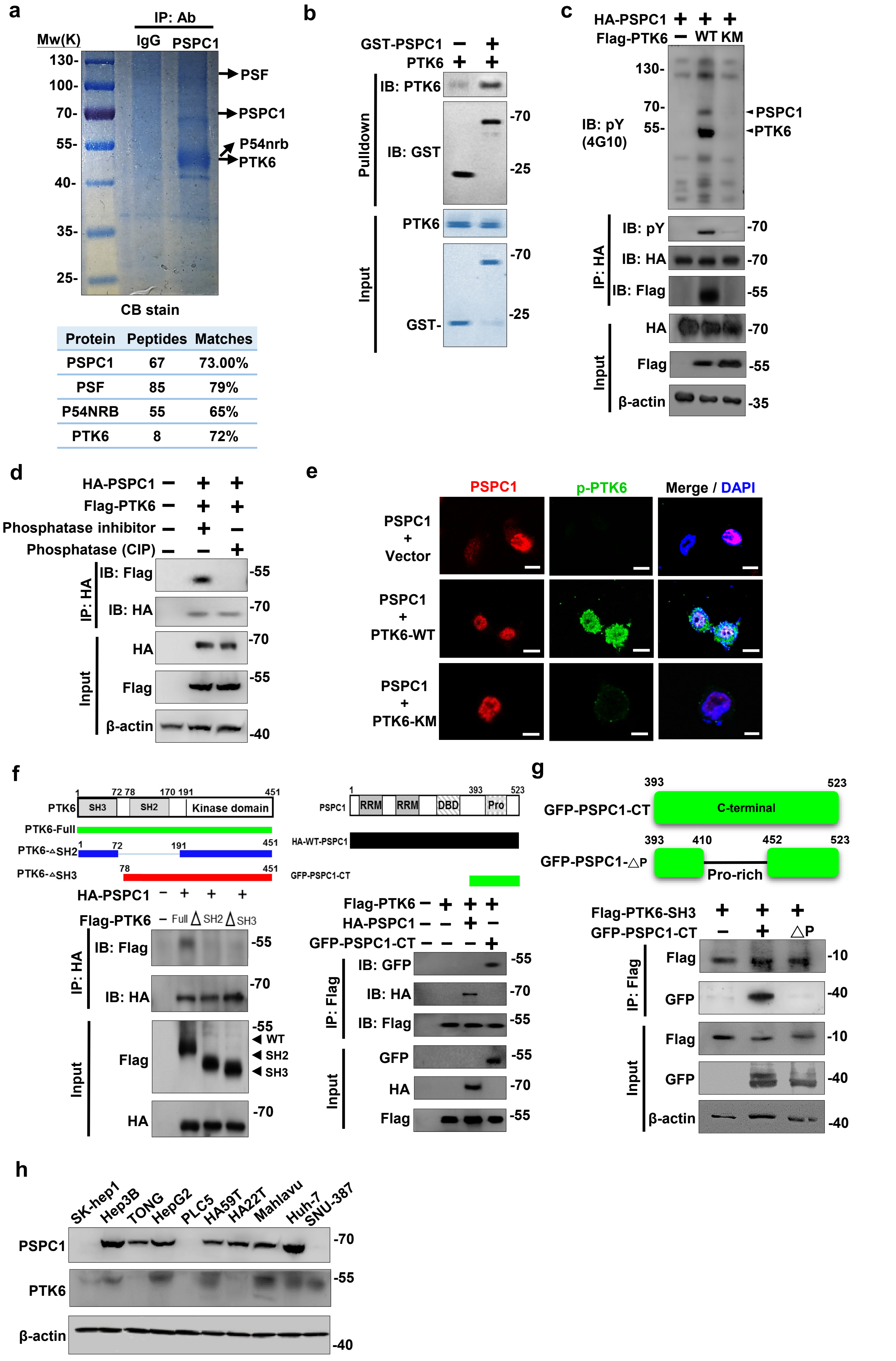

Protein	Peptides	Matches
PSPC1	67	73.00%
PSF	85	79%
P54NRB	55	65%
PTK6	8	72%

Supplementary Figure 1 Interactions between PSPC1 and PTK6 via the C-terminal proline-rich domain of PSPC1 and the SH2 and SH3 domains of PTK6.

a Coomassie blue staining of the PSPC1 immunoprecipitated (IP) complex of Huh-7 cell lysates with anti-PSPC1 antibody separated by SDS-PAGE (upper panel). The three bands were excised and subjected to LC-MS/MS analysis. Preimmune IgG served as an IP control, and four interacting proteins were identified including matching percentage and the number of peptides (lower panel). Detailed information on IP peptides is included in Supplementary Table 1. **b** Direct interaction of PSPC1 and PTK6 by GST pull-down assay performed by using purified recombinant GST-PSPC1 and the active form of recombinant protein PTK6, followed by IB with anti-PTK6 antibody (upper panel). Coomassie blue staining indicates equal inputs of GST-PSPC1 and PTK6 (lower panel). **c** PSPC1 interacting with PTK6 is dependent on tyrosine kinase phosphorylation demonstrated by IP/Western blotting analysis with 4G10 antibody and indicated protein antibodies. HA-tagged PSPC1, Flag-tagged PTK6 WT and Flag-tagged PTK6 kinase-dead (KM) proteins were expressed in 293T cells. **d** Treatment with alkaline phosphatase (CIP) in contrast to treatment with a phosphatase inhibitor disrupted HA-tagged PSPC1 and Flag-tagged PTK6 protein interaction demonstrated via IP/Western analysis of SK-hep1 cells lysates. **e** IF analysis of subcellular localization of PSPC1 and PTK6 by expressing HA-tagged PSPC1 with wild-type or kinase-dead (KM) mutants of Flag-tagged PTK6 in SK-hep1 cells. Colors are PSPC1 (red), PTK6 (green) and nuclei (DAPI, blue). The scale bar represents 20 μm. **f, g,** Protein interaction domains of PSPC1 and PTK6. (**f**) Left: Top, schematic domain deletion constructs of PTK6. Bottom, the SH2 and SH3 domains are required for PSPC1 and PTK6 interaction demonstrated by expressing HA-tagged PSPC1 and coexpressing full-length and deletion constructs of Flag-tagged PTK6 in 293T cells and IP/Western analysis. Right: Top, schematic domain deletion constructs of PSPC1. Bottom, the proline-rich C-terminal domain of PSPC1 is required for PSPC1 and PTK6 interaction demonstrated by IP/Western analysis of 293T cells expressing Flag-tagged PTK6 and coexpressing full-length or GFP-tagged PSPC1 C-terminal (CT). **g** Top, schematic domain deletion constructs of GFP-tagged PSPC1 C-terminus (CT). The proline-rich C-terminal domain of PSPC1 is essential for PSPC1 and PTK6 interaction demonstrated by IP/Western analysis of 293T cells expressing Flag-tagged PTK6-SH3 and coexpressing GFP-tagged PSPC1-CT and the proline deletion construct of GFP-tagged PSPC1-CT. **h** Endogenous expression of PSPC1 and PTK6 proteins in ten hepatocellular carcinomas cell lines determined by Western blotting analysis. β-actin is the loading and internal control.

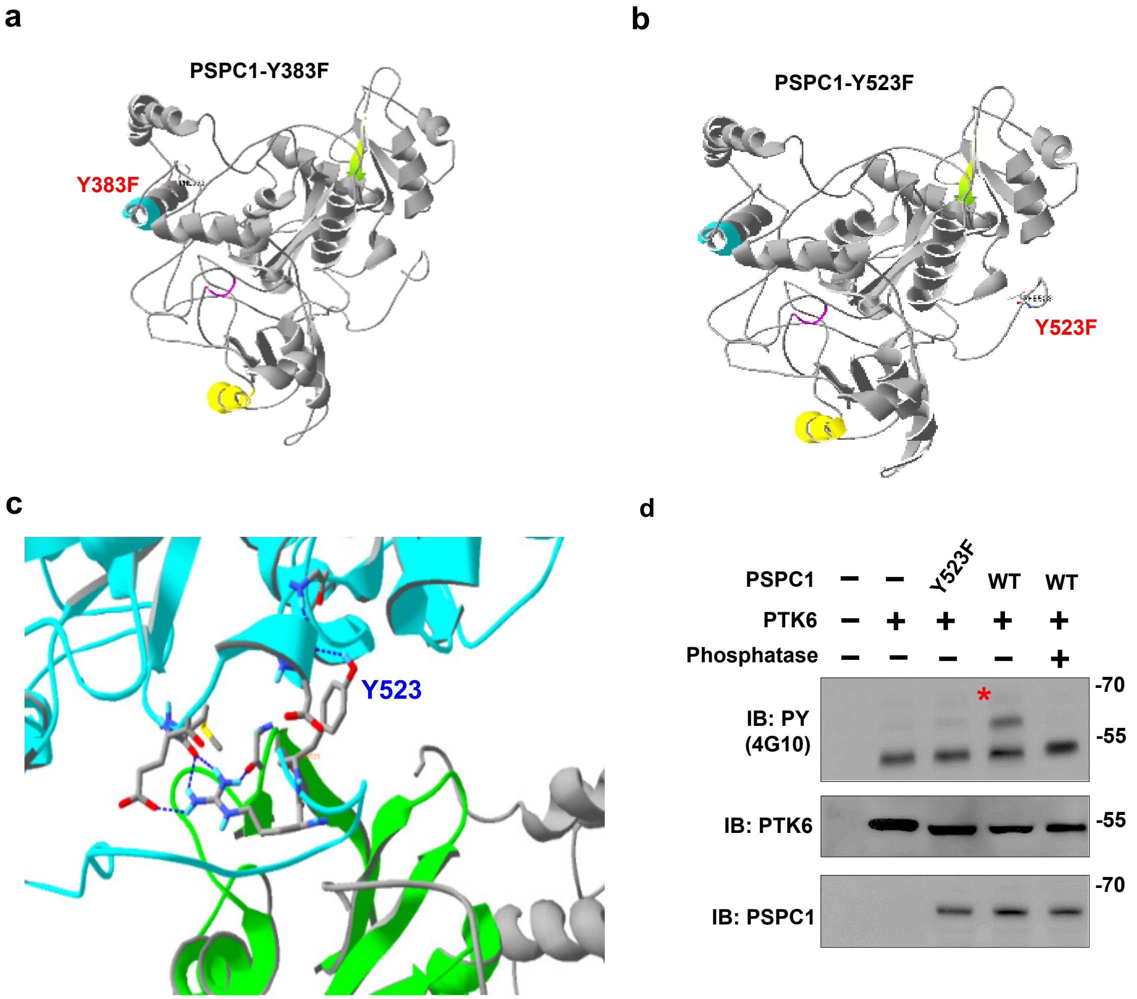

Supplementary Figure 2 The simulated protein structure prediction of the dynamic interactions of PSPC1, PSPC1-Y523F and PTK6.

a-c The predicted 3D protein structure of PSPC1 with tyrosine to phenylalanine mutations at Y383 (**a**) and Y523 (**b**) as well as the predicted docking interface of PSPC1 and PTK6 (**c**). The Y383F and Y523F mutations of PSPC1 have similar protein 3D structure changes (labeled in green/yellow/purple/cyan) compared to wild-type PSPC1. (**c**) The interface of the PSPC1 and PTK6 interaction. The left side (Caribbean blue color) is PSPC1, the right side (green color) is PTK6, and the blue dotted lines represent the hydrogen bonds near the interface. **d** *In vitro* kinase assays were performed by using anti-pY (4G10), anti-PSPC1 and anti-PTK6 antibodies. Purified recombinant PSPC1, PSPC1-YF (Y523F) and PTK6 proteins were incubated in kinase buffer. Asterisks denote the tyrosine-phosphorylated protein.

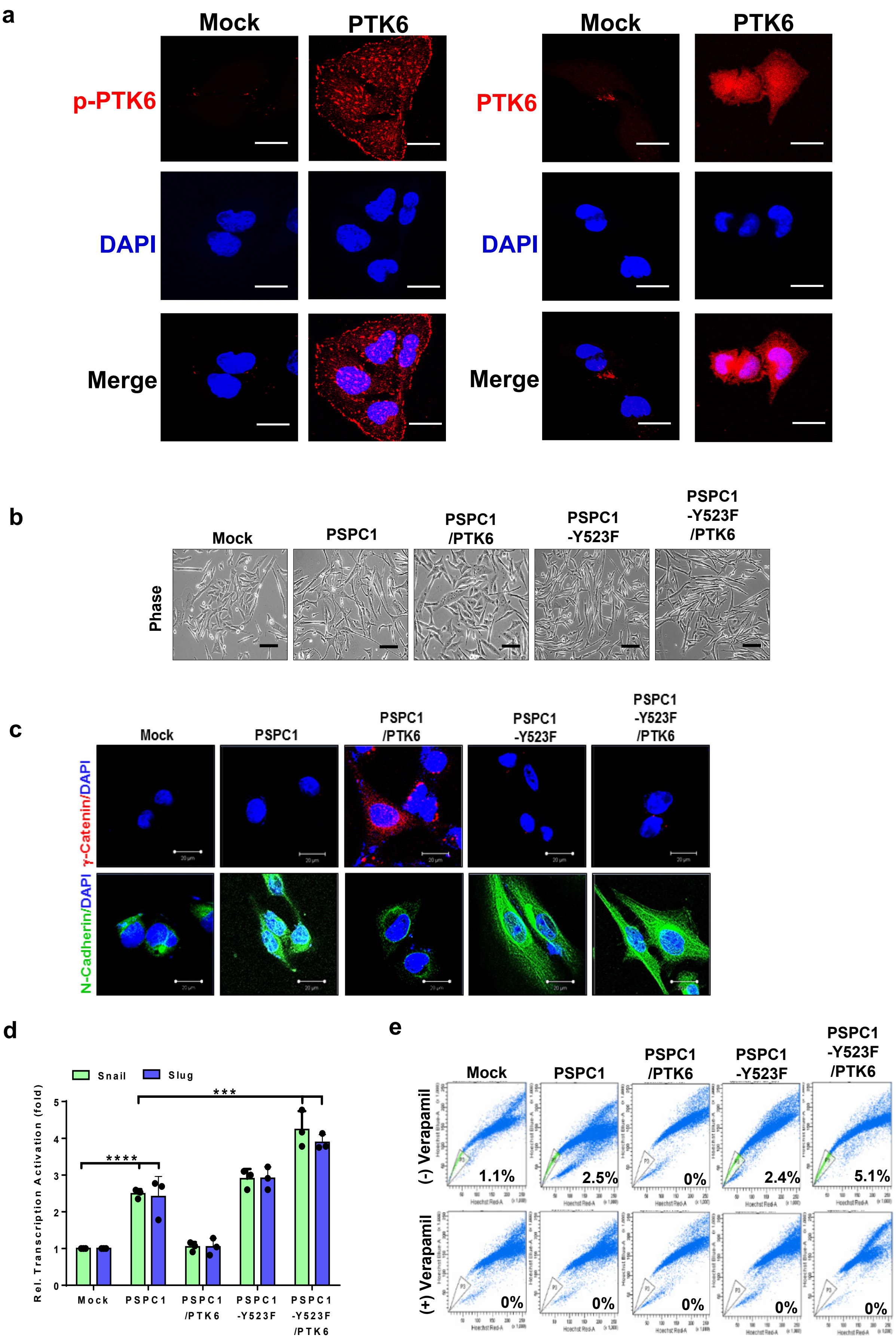

a
Mock
PTK6
p-PTK6
DAPI
Merge
Mock
PTK6
PTK6
DAPI
Merge
b
Mock
PSPC1
PSPC1 /PTK6
PSPC1 -Y523F
PSPC1 -Y523F /PTK6
Phase
c
Mock
PSPC1
PSPC1 /PTK6
PSPC1 -Y523F
PSPC1 -Y523F /PTK6
γ-Catenin/DAPI
N-Cadherin/DAPI
d
Snail
Slug
Rel. Transcription Activation (fold)
****
***
Mock
PSPC1
PSPC1 /PTK6
PSPC1 -Y523F
PSPC1 -Y523F /PTK6
e
Mock
PSPC1
PSPC1 /PTK6
PSPC1 -Y523F
PSPC1 -Y523F /PTK6
(-) Verapamil
(+) Verapamil
1.1%
2.5%
0%
2.4%
5.1%
0%
0%
0%
0%
0%

Supplementary Figure 3 PSPC1 deficient, upregulation and expression of the PSPC1-Y523F mutant modulated PTK6 from nuclear sequestration to synergize the oncogenic effects of PSPC1 and cytoplasmic PTK6.

a Immunofluorescence analysis for the expression of phospho-PTK6 (p-PTK6) and PTK6. SK-hep1 (deficient of PSPC1) cells were transfected with the indicated plasmids. Colors are p-PTK6 and PTK6 in red, and nuclei with DAPI in blue. The scale bar represents 25 μm. **b** Analysis of cell morphology in phase image of the indicated PSPC1/PTK6 SK-hep1 cell transfectants. The scale bar represents 50 μm. **c** The expression of epithelial (r-catenin) and mesenchymal markers by IF in the indicated PSPC1/PTK6 SK-hep1 cell transfectants. Colors are as follows: r-catenin (red), N-cadherin (green) and nuclei (DAPI, blue). The scale bar represents 20 μm. **d** Luciferase activity of Snail and Slug promoter reporters after cotransfection of different expression constructs. The luciferase activity/β-galactosidase of 293T cells cotransfected with the pcDNA3 control vector was used as the baseline control. Data are represented as the mean ± SEM (n=3). All data statistics were based on *$p < 0.05$, **$p < 0.01$, and ***$p < 0.001$ calculated by Brown-Forsythe test. **e** Flow cytometry analyses of the side population fraction of PSPC1/PTK6 SK-hep1 cell transfectants in the presence and absence of Verapamil.

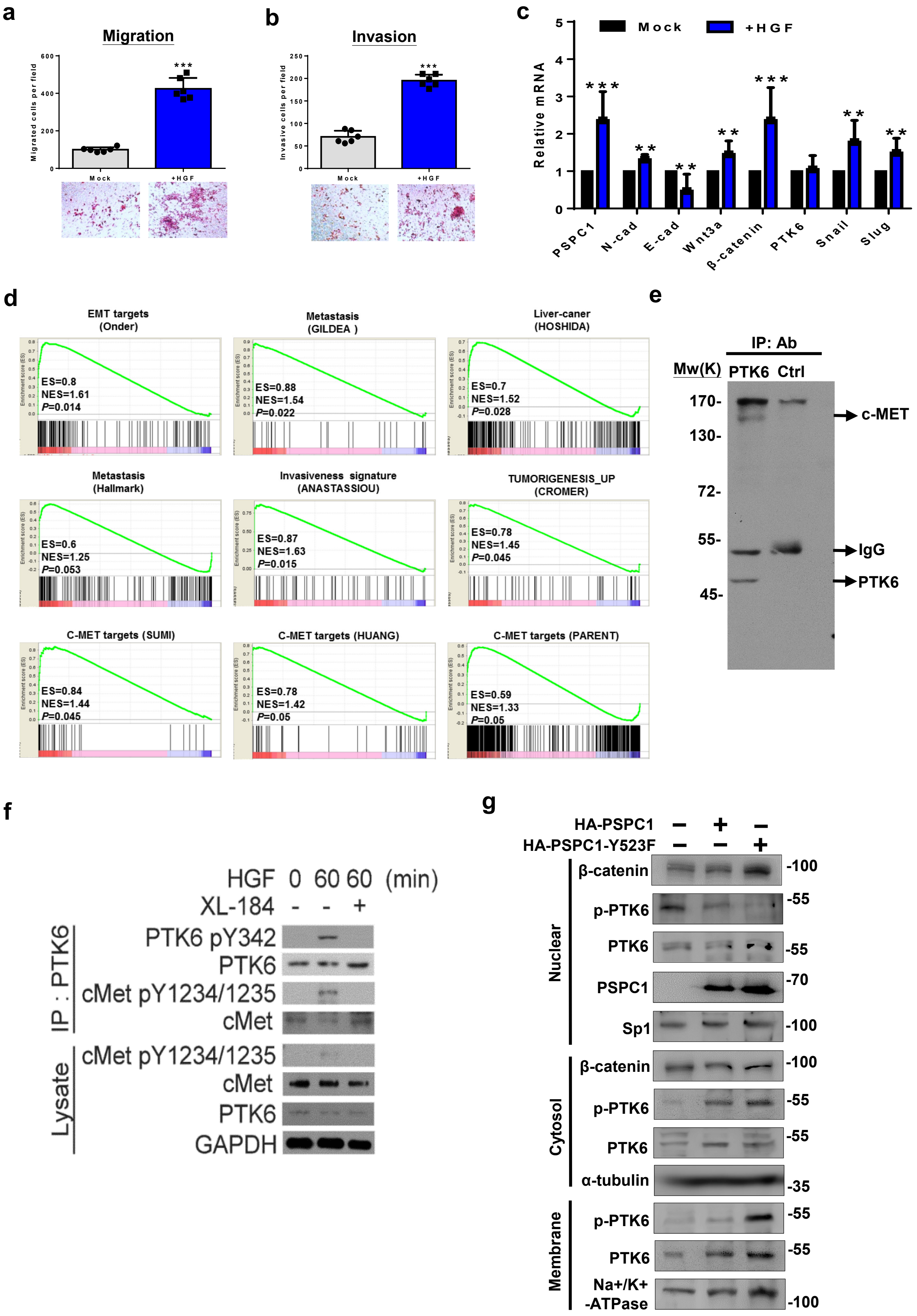
a
Migration
Migrated cells per field
600
400
200
0
***
Mock
+HGF
b
Invasion
Invasive cells per field
250
200
150
100
50
0
***
Mock
+HGF
c
Mock
+HGF
Relative mRNA
5
4
3
2
1
0
***
**
**
**
***
**
**
PSPC1
N-cad
E-cad
Wnt3a
β-catenin
PTK6
Snail
Slug
d
EMT targets (Onder)
ES=0.8
NES=1.61
P=0.014
Metastasis (GILDEA)
ES=0.88
NES=1.54
P=0.022
Liver-caner (HOSHIDA)
ES=0.7
NES=1.52
P=0.028
Metastasis (Hallmark)
ES=0.6
NES=1.25
P=0.053
Invasiveness signature (ANASTASSIOU)
ES=0.87
NES=1.63
P=0.015
TUMORIGENESIS_UP (CROMER)
ES=0.78
NES=1.45
P=0.045
C-MET targets (SUMI)
ES=0.84
NES=1.44
P=0.045
C-MET targets (HUANG)
ES=0.78
NES=1.42
P=0.05
C-MET targets (PARENT)
ES=0.59
NES=1.33
P=0.05
e
IP: Ab
Mw(K)
PTK6
Ctrl
170-
130-
72-
55-
45-
c-MET
IgG
PTK6
f
HGF 0 60 60 (min)
XL-184 - - +
IP : PTK6
PTK6 pY342
PTK6
cMet pY1234/1235
cMet
Lysate
cMet pY1234/1235
cMet
PTK6
GAPDH
g
HA-PSPC1 – + –
HA-PSPC1-Y523F – – +
Nuclear
β-catenin
p-PTK6
PTK6
PSPC1
Sp1
-100
-55
-55
-70
-100
Cytosol
β-catenin
p-PTK6
PTK6
α-tubulin
-100
-55
-55
-35
Membrane
p-PTK6
PTK6
Na+/K+ -ATPase
-55
-55
-100

Supplementary Figure 4 HGF-activated oncogenic PSPC1/Wnt3a/c-Met/p-PTK6 axis augments downstream oncogenic signaling.

a, b Migration (**a**) and Matrigel cell invasion (**b**) assays of Huh7 cells treated with 10 ng/ml HGF for 48 h. Data represent the mean ± SEM (n=6). Data statistics are based on *p < 0.05, **p < 0.01, and ***p < 0.001 calculated by one-way ANOVA with Brown-Forsythe test. **c** mRNA expression of PSPC1/PTK6/Wnt pathway-related genes under HGF stimulation with PSPC1, E-cadherin, N-cadherin, Wnt3a, β-catenin, PTK6, Snail and Slug normalized with GAPDH in Huh-7 cells detected by qRT-PCR. Data are the means ± SEM (n=3). All data statistics were based on: *p < 0.05, **p < 0.01, and ***p < 0.001 calculated by Student's t-test compared to the Mock control. **d** GSEA profiling of metastasis, EMT and HGF/c-MET signaling in HGF-treated Huh-7 cells after transcriptome and GSEA analysis. ES, enrichment score; NES: normalized enrichment score in HGF high compared to control population. **e** Interaction of PTK6 with c-Met in HGF-treated Huh-7 cell lysate analyzed by IP/Western with anti-PTK6 antibody or control mouse IgG. **f** IP/Western analysis for detection of PTK6 phosphorylation of pY342 and activated phospho-c-Met at pY1234/1235 upon HGF stimulation. HGF-treated Huh-7 cells pretreated with 100 nM XL-184 (c-Met inhibitor) 24 h before serum starvation and then stimulated with 50 ng/ml HGF for 60 min. **g** Dynamic PSPC1 and PSPC1-Y523F interactions with PTK6 and β-catenin altering their subcellular localization in SNU-387 cells demonstrated by Western blotting analysis with corresponding markers for including nuclear (Sp1), cytosolic (α-tubulin) and membrane (Na+/K+-ATPase) localizations.

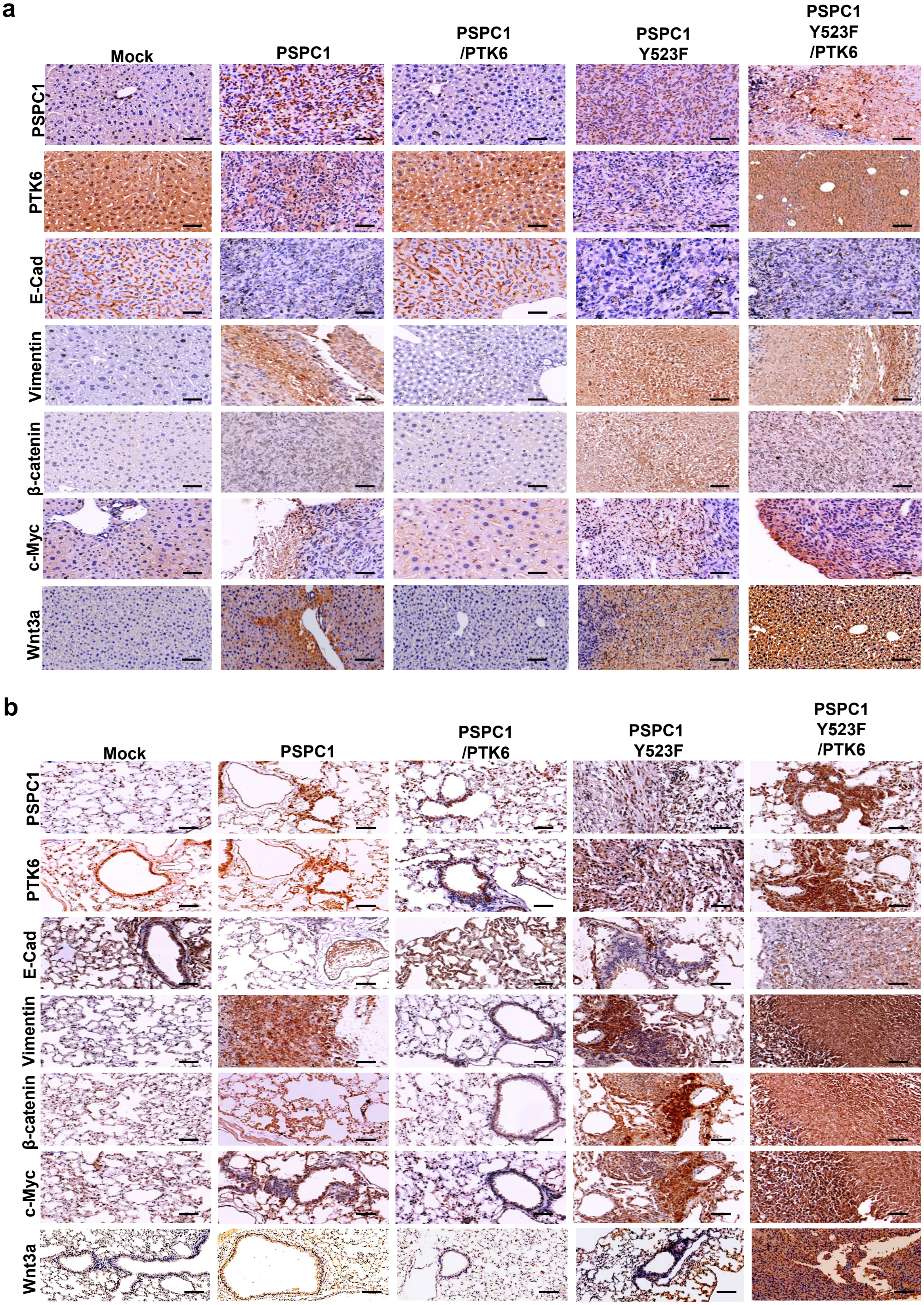

Supplementary Figure 5 The PSPC1/PTK6/β-catenin axis is critical for tumor growth and metastasis in HCC.

a, b Representative IHC staining of PSPC1, PTK6, E-cadherin, vimentin, β-catenin, c-Myc and Wnt3a from tumor tissues in the liver (**a**) and lung (**b**) isolated from the mice of orthotopic murine hepatocellular carcinoma model injected with different PSPC1/PTK6 SK-hep1 cell transfectants. Scale bars, 50 μm.

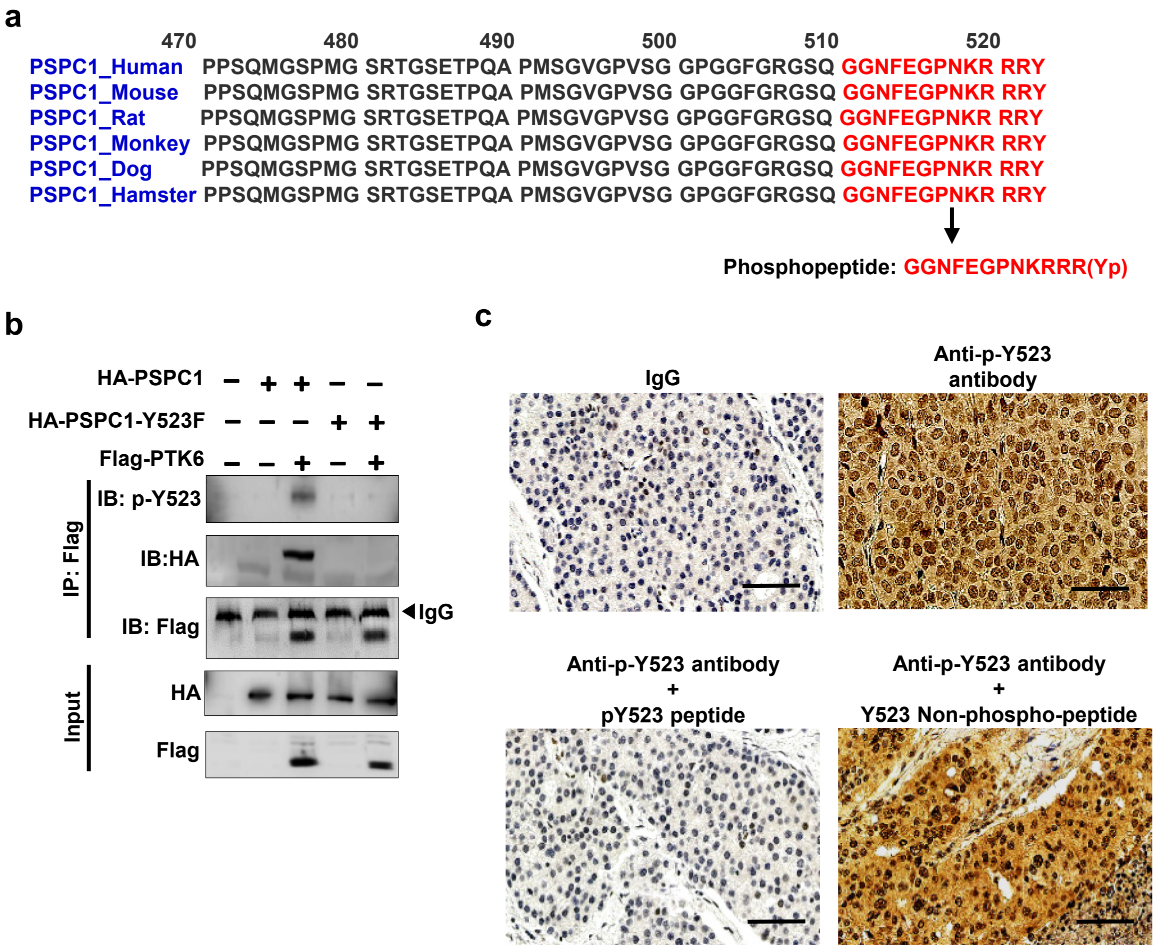

Supplementary Figure 6 Validation of the antibody specificity and the expression intensity of p-Y523-PSPC1 in 215 human HCC tissues.

a Conserved sequences of mammalian PSPC1 orthologs surrounding the Y523 residue of human PSPC1. The arrow indicates that the phosphopeptide (red text) was used as the antigen to raise a phospho-Y523-PSPC1-specific polyclonal antibody. **b** IP/Western analysis using the anti-phospho-PSPC1 Y523 antibody (PSPC1 p-Y523) in 293T cells transfected with PSPC1, PSPC1-Y523F and PTK6 constructs. **c** Validating the specificity of the anti-phospho-PSPC1 Y523 antibody for IHC staining of HCC tumor tissues. Representative IHC images of human HCC tumor tissue stained with the anti-phospho-PSPC1 Y523 antibody competing with the corresponding phosphopeptides and nonphosphopeptides. Scale bar = 25 μm.

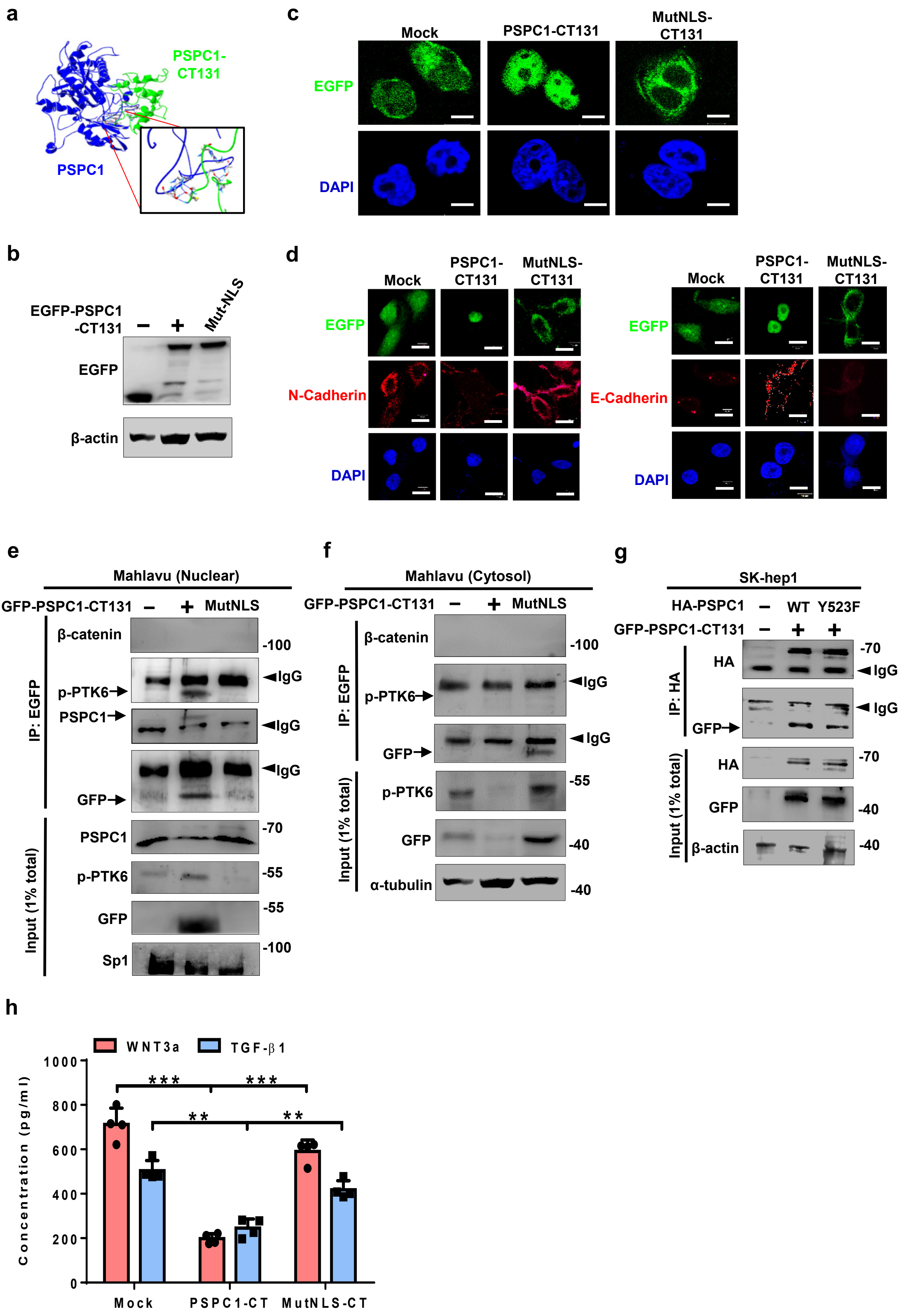

a
PSPC1-CT131
PSPC1
c
Mock
PSPC1-CT131
MutNLS-CT131
EGFP
DAPI
b
EGFP-PSPC1-CT131
−
+
Mut-NLS
EGFP
β-actin
d
Mock
PSPC1-CT131
MutNLS-CT131
EGFP
N-Cadherin
DAPI
E-Cadherin
e
Mahlavu (Nuclear)
GFP-PSPC1-CT131 − + MutNLS
IP: EGFP
β-catenin
p-PTK6
PSPC1
GFP
IgG
-100
Input (1% total)
PSPC1
p-PTK6
GFP
Sp1
-70
-55
-55
-100
f
Mahlavu (Cytosol)
GFP-PSPC1-CT131 − + MutNLS
IP: EGFP
β-catenin
p-PTK6
GFP
IgG
-100
Input (1% total)
p-PTK6
GFP
α-tubulin
-55
-40
-40
g
SK-hep1
HA-PSPC1 − WT Y523F
GFP-PSPC1-CT131 − + +
IP: HA
HA
GFP
IgG
-70
Input (1% total)
HA
GFP
β-actin
-70
-40
-40
h
WNT3a
TGF-β1
Concentration (pg/ml)
0
200
400
600
800
1000
***
***
**
**
Mock
PSPC1-CT
MutNLS-CT

Supplementary Figure 7 PSPC1-CT131 is a dual inhibitor suppressing PSPC1- and PTK6-mediated reciprocal PTK6 and β-catenin subcellular translocations as well as autocrine signaling of Wnt3a and TGF-β1.

a The structural docking of PSPC1-CT131 (green) with PSPC1 (blue). Structures were predicted by using I-TASSER. The protein-protein docking predictions were performed by using ClusPro. **b** Expression of EGFP-PSPC1-CT131 and Mut-NLS-CT131 (nuclear localization sequence (NLS) mutant of PSPC1-CT) in Mahlavu cells demonstrated by Western blotting analysis. **c** Subcellular localization of enhanced green fluorescent protein (EGFP)-conjugated PSPC1-CT131 and Mut-NLS-CT131 in Mahlavu cells. The scale bar represents 10 μm. **d** Expression of PSPC1-CT131 but not Mut-NLS-CT enhanced the epithelial phenotype and E-cadherin expression and reduced the mesenchymal phenotype and N-cadherin expression in Mahlavu cells determined by IF analysis. Colors are N-Cadherin and E-Cadherin in red, EGFP in green and nuclei with DAPI in blue. The scale bar represents 10 μm. **e, f,** Interactions and subcellular localization of PSPC1-CT131 with PSPC1 and p-PTK6 in the nucleus (**e**) but not the cytoplasm (**f**) in Mahlavu cells demonstrated by IP/Western blotting analysis. **g** Interaction of PSPC1-CT131 with wild-type PSPC1 and Y523F mutant PSPC1 in SK-hep1 cells shown by IP/Western blotting analysis. **h** Expression of PSPC1-CT131 but not Mut-NLS-CT in Mahlavu cells decreased the levels of cytokines TGF-β1 and Wnt3a in the conditioned medium demonstrated by ELISA. Data represent the mean ± SEM (n=4). All data statistics were based on: *$p < 0.05$, **$p < 0.01$, and ***$p < 0.001$ calculated by one-way ANOVA with Brown-Forsythe test.

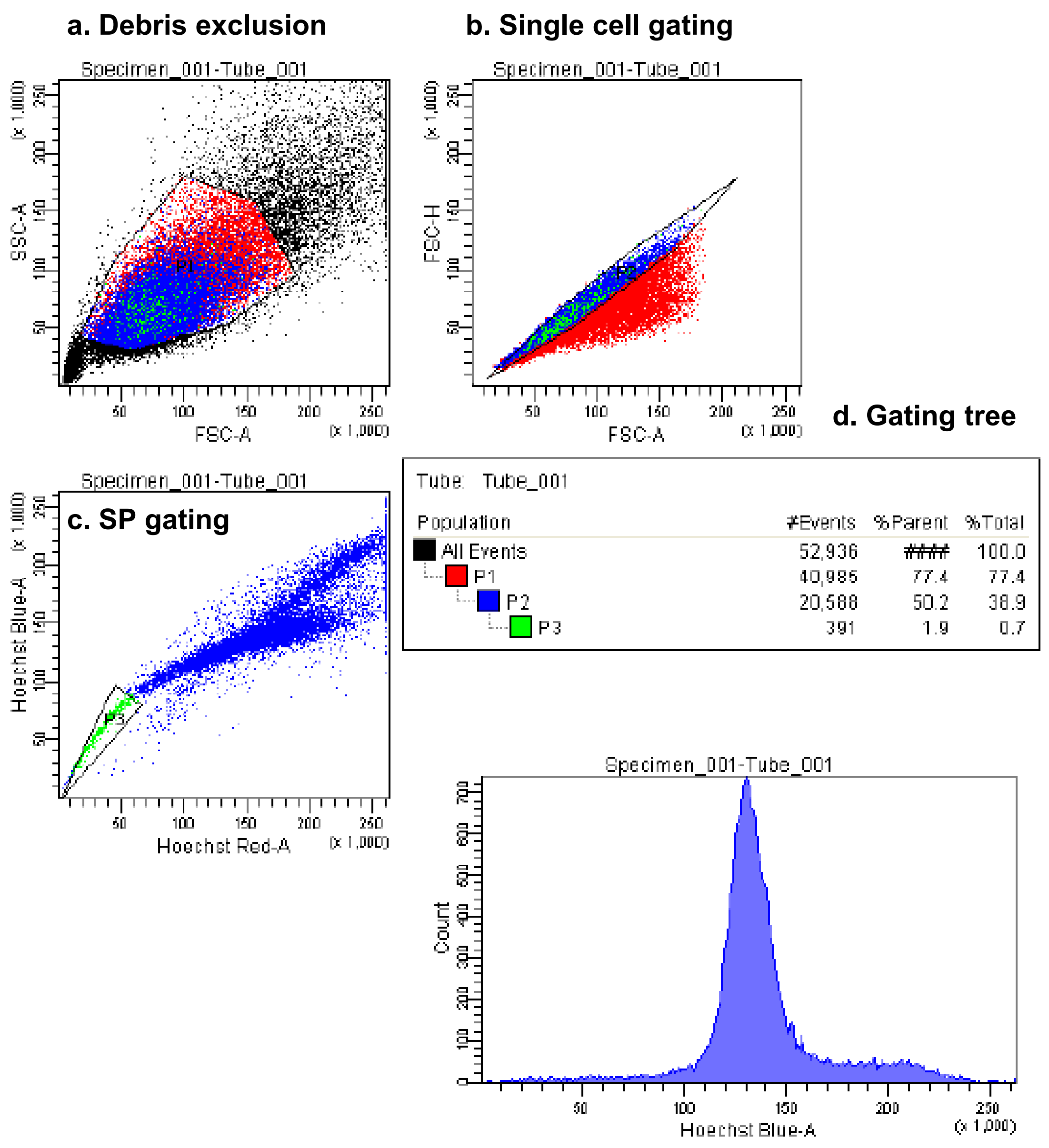

Supplementary Figure 8 Gating Strategy for side population (SP) Analysis

a Cells are isolated from debris on the flow-cytometric profile based on the Forward Scatter (FSC) and Side Scatter (SSC). **b** Cell aggregates and doublets are gated out and display on the SSC area (SSC-A) v.s height (SSC-H) dot plot. **c** Side population cells are recognized as a dim tail extending toward the lower Hoechst Blue signal (P3). **d** The gating tree indicates the sequential procedure applied to select out the final population for SP discrimination and the percentage of cells (gated events) from each gating step. “% Parent” indicates the percentage of gated events relative to the preceding gate, and “% Total” indicates the percentage of gated events relative to all events recorded.

Supplementary Table 1 Identification of peptide fragments after IP of PSPC1-interacting protein complex of Huh7 cells and LC/MS studies.

Supplementary Table 1: Identification of peptide fragments after IP of PSPC1-interacting protein complex of Huh7 cells and LC/MS studies										
Database : NCBI and SwissProt										
gi|109240550 Mass: 58706 Score: 42348 Matches: 2138(1560) Sequences: 44(32) emPAI: 1241.36										
**paraspeckle component 1 [Homo sapiens]**										
**Query**	Observed	Mr(expt)	Mr(calc)	ppm	Miss	Score	Expect	Rank	Unique	Peptide
**210**	390.163	778.311	778.31	0.42	0	42	0.0044	1	U	R.MGDMGPR.G
**253**	398.16	794.305	794.305	-0.34	0	-34	0.022	1	U	R.MGDMGPR.G
**298**	408.24	814.466	814.466	0.34	1	35	0.14	1		R.RLEELR.N
**330**	415.728	829.441	829.441	0.67	1	20	5.5	3		R.RQEELR.R
**351**	418.197	834.38	834.379	0.39	0	35	0.1	1		K.ALDEMEK.Q
**437**	426.195	850.375	850.374	0.62	0	-27	0.4	1		K.ALDEMEK.Q
**542**	436.746	871.478	871.476	2.17	0	48	0.0072	1		K.AVVVVDDR.G
**653**	453.258	904.502	904.502	-0.13	1	39	0.037	1		K.SFLKPGEK.T
**690**	456.238	910.462	910.462	-0.25	1	22	1.6	1		K.EREQPPR.F
**926**	480.274	958.533	958.534	-0.33	0	53	0.0026	1		K.AELDGTILK.S
**1026**	484.259	966.504	966.504	0.21	1	29	0.32	1		R.DRGFGFIR.L
**1181**	493.778	985.542	985.542	-0.16	2	39	0.052	1		R.RQEELRR.L
**1329**	510.245	1018.48	1018.48	-0.22	0	-64	1.60E-04	1		K.LEAEMEAAR.H
**1421**	518.242	1034.47	1034.47	-1.08	0	65	9.70E-05	1		K.LEAEMEAAR.H
**1637**	362.54	1084.6	1084.6	-0.08	1	-36	0.087	1		K.AVVVVDDRGR.A
**1640**	543.308	1084.6	1084.6	2.41	1	55	0.00091	1		K.AVVVVDDRGR.A
**1746**	366.188	1095.54	1095.54	-0.28	1	42	0.027	1		R.HREQEELR.R
**1748**	548.778	1095.54	1095.54	0	1	-21	3.3	2		R.HREQEELR.R
**1938**	558.314	1114.61	1114.61	-0.32	0	65	0.00017	1		R.FATHGAALTVK.N
**2038**	559.79	1117.57	1117.57	-0.15	2	-25	1.9	1		R.RREEEMIR.H
**2039**	373.529	1117.57	1117.57	0.22	2	42	0.034	1		R.RREEEMIR.H
**2118**	378.861	1133.56	1133.56	-0.04	2	-29	0.69	1		R.RREEEMIR.H
**2121**	567.788	1133.56	1133.56	0.14	2	-27	1.3	1		R.RREEEMIR.H
**2229**	575.284	1148.55	1148.55	-0.1	1	29	0.82	1		R.WKALDEMEK.Q
**2424**	596.273	1190.53	1190.53	-1.04	0	60	0.00025	1	U	R.GSQGGNFEGPNK.R
**2430**	596.764	1191.51	1191.52	-1.25	0	-45	0.0058	1	U	R.GSQGGNFEGPNK.R
**2431**	596.765	1191.52	1191.52	0.08	0	-55	0.00065	1	U	R.GSQGGNFEGPNK.R
**2450**	398.867	1193.58	1193.58	-0.62	0	-45	0.014	1		R.HEHQLMLMR.Q
**2552**	601.841	1201.67	1201.67	-0.13	1	41	0.03	1		K.AELDGTILKSR.P
**2553**	401.563	1201.67	1201.67	0.66	1	-23	2	2		K.AELDGTILKSR.P
**2620**	404.199	1209.57	1209.57	-0.39	0	-38	0.053	1		R.HEHQLMLMR.Q
**2665**	404.533	1210.58	1210.56	14.8	0	-22	2.4	1		R.HEHQLMLMR.Q 2666
**2724**	409.526	1225.56	1225.57	-11.16	0	-35	0.099	1		R.HEHQLMLMR.Q
**2900**	416.539	1246.6	1246.6	-1.76	1	-22	2.9	1		K.ALDEMEKQQR.E
**2904**	624.797	1247.58	1247.58	-1.79	1	46	0.0088	1		K.ALDEMEKQQR.E
**3128**	638.814	1275.61	1275.61	-0.25	1	54	0.0026	1		K.EKLEAEMEAAR.H
**3396**	645.354	1288.69	1288.69	-0.06	1	64	0.00019	1		K.GFVEFAAKPPAR.K
**3469**	431.543	1291.61	1291.61	-0.96	1	-34	0.2	1		K.EKLEAEMEAAR.H
protein-tyrosine kinase 6 isoform 1 [Homo sapiens]										
**Query**	Observed	Mr(expt)	Mr(calc)	ppm	Miss	Score	Expect	Rank	Unique	Peptide
**925**	450.779	899.544	899.544	-0.15	0	55	0.00084	1	U	K.GSLLELLR.D
**1769**	514.266	1026.52	1026.52	0.08	0	30	0.27	1	U	K.YVGLWDFK.S
**1795**	515.769	1029.52	1029.52	-0.41	0	47	0.0072	1	U	K.WTAPEALSR.G
**5593**	517.278	1548.81	1548.81	-0.93	1	41	0.041	1	U	R.VSEKPSADYVLSVR.D
**625**	450.779	1613.8	1613.8	-0.11	0	55	0.04418	1	U	K.GSLLELLLR.D
**1562**	514.266	1808.88	1808.89	0.08	0	25	0.029948	1	U	K.YVVGSLWDFK.S
**1425**	515.769	2003.97	2003.97	-0.32	0	41	0.015716	1	U	K.WTAAPEALSSR.G
**6712**	517.278	2199.05	2199.05	-0.12	1	48	0.001484	1	U	R.VCSEKPSADYEVLSVR.D
non-POU domain-containing octamer-binding protein isoform 1 [Homo sapiens]										
**Query**	Observed	Mr(expt)	Mr(calc)	ppm	Miss	Score	Expect	Rank	Unique	Peptide
**66**	376.203	750.391	750.391	0.27	0	30	0.3	1	U	K.TFNLEK.Q 65
**263**	415.728	829.442	829.441	1.47	1	21	4.4	2		R.RQEELR.R
**270**	417.226	832.437	832.436	1	0	34	0.19	1		K.ALIEMEK.Q 269
**282**	418.73	835.444	835.444	0.56	0	31	0.24	1		K.GIVEFSGK.P
**321**	425.223	848.431	848.431	-0.09	0	-22	2.6	1		K.ALIEMEK.Q
**443**	443.753	885.491	885.492	-0.84	0	43	0.017	1		R.AVVIVDDR.G 444
**528**	456.238	910.462	910.462	0.09	1	23	1.3	1		K.EREQPPR.F
**633**	470.255	938.496	938.497	-1.37	1	36	0.058	1		K.DKGFGFIR.L
**971**	493.778	985.542	985.542	-0.02	2	24	1.6	1		R.RQEELRR.M
**1341**	536.775	1071.53	1071.53	-0.22	0	55	0.001	1		R.AAPGAEFAPNK.R
**1365**	540.247	1078.48	1078.48	0.4	0	52	0.0017	1		K.LEMEMEAAR.H
**1485**	544.277	1086.54	1086.54	0.47	0	-54	0.0016	1		K.VELDNMPLR.G
**1510**	548.244	1094.47	1094.47	-0.54	0	-35	0.075	1		K.LEMEMEAAR.H
**1553**	551.781	1101.55	1101.55	-2.02	0	-56	0.0011	1		K.VELDNMPLR.G
**1612**	556.241	1110.47	1110.47	-1.01	0	-47	0.0039	1		K.LEMEMEAAR.H
**1859**	380.502	1138.48	1138.49	-0.84	1	25	0.78	1		R.RQQEEMMR.R
**1888**	381.877	1142.61	1142.61	-0.65	1	24	1.8	1		K.AGEVFIHKDK.G
**2079**	590.79	1179.57	1179.56	0.9	0	-39	0.051	1		R.HEHQVMLMR.Q
**2139**	596.295	1190.57	1190.59	-10.24	0	-40	0.061	1		R.FACHSASLTVR.N
**2176**	598.787	1195.56	1195.56	-0.38	0	-36	0.1	1		R.HEHQVMLMR.Q
**2432**	614.825	1227.64	1227.64	-0.82	1	-46	0.01	1		R.AAPGAEFAPNKR.R 2431
**2435**	615.316	1228.62	1228.62	-1.91	1	80	4.50E-06	1		R.AAPGAEFAPNKR.R
**2458**	616.343	1230.67	1230.67	-1.36	1	47	0.0094	1		K.GIVEFSGKPAAR.K
**splicing factor, proline- and glutamine-rich [Homo sapiens]**										
**Query**	Observed	Mr(expt)	Mr(calc)	ppm	Miss	Score	Expect	Rank	Unique	Peptide
**24**	358.221	714.428	714.428	0.21	0	51	0.0035	1		R.ALAEIAK.A
**261**	394.237	786.459	786.46	-0.6	0	-53	0.0025	1		K.ANLSLLR.R
**531**	425.737	849.459	849.46	-0.41	0	38	0.047	1		K.GIVEFASK.P
**535**	426.195	850.374	850.374	0.26	0	36	0.048	1		K.SLDEMEK.Q
**547**	427.25	852.486	852.486	0.39	1	34	0.057	1		K.GKGFGFIK.L
**618**	435.186	868.358	868.357	0.59	0	51	0.00081	1		R.MGYMDPR.E
**684**	441.714	881.414	881.413	0.97	0	44	0.0083	1		K.ISDSEGFK.A
**701**	443.183	884.352	884.352	-0.3	0	-49	0.00062	1		R.MGYMDPR.E
**717**	443.753	885.492	885.492	-0.02	0	40	0.031	1		R.AVVIVDDR.G
**978**	469.21	936.404	936.404	-0.06	0	-24	1.1	1		R.EEEMMIR.Q 977
**1064**	476.697	951.379	951.379	0.35	0	34	0.038	1		R.EMEEQMR.R
**1071**	477.207	952.399	952.399	-0.49	0	24	0.63	1		R.EEEMMIR.Q
**1280**	492.692	983.369	983.369	-0.18	0	-23	0.23	1		R.EMEEQMR.R 1281
**1762**	532.24	1062.46	1062.47	-0.21	0	-37	0.043	1		K.AELDDTPMR.G
**1989**	547.259	1092.5	1092.51	-1.76	1	38	0.056	1		R.REEEMMIR.Q 1991
**1990**	365.176	1092.51	1092.51	-0.54	1	-25	1.3	1		R.REEEMMIR.Q
**2041**	550.315	1098.61	1098.61	-0.19	1	48	0.0046	1		R.AVVIVDDRGR.S
**2107**	555.257	1108.5	1108.5	-1.44	1	-31	0.22	1		R.REEEMMIR.Q
**2108**	555.257	1108.5	1108.5	-0.56	1	-34	0.13	1		R.REEEMMIR.Q 2109
**2313**	568.761	1135.51	1135.51	0.09	0	-32	0.23	1	U	R.GMGPGTPAGYGR.G
**2418**	381.881	1142.62	1142.62	0.26	0	-47	0.0094	1		R.FATHAAALSVR.N
**2494**	385.217	1152.63	1152.63	-0.1	1	-35	0.085	1		K.GFGFIKLESR.A 2493
**2495**	577.322	1152.63	1152.63	-0.01	1	40	0.027	1		K.GFGFIKLESR.A
**2576**	583.281	1164.55	1164.55	-0.24	1	48	0.0076	1		R.WKSLDEMEK.Q 2578
**2577**	389.19	1164.55	1164.55	-0.1	1	-37	0.085	1		R.WKSLDEMEK.Q
**2676**	591.278	1180.54	1180.54	-1	1	-31	0.25	1		R.WKSLDEMEK.Q
**2677**	394.521	1180.54	1180.54	-0.81	1	-29	0.49	1		R.WKSLDEMEK.Q
**2865**	404.202	1209.58	1209.59	-0.4	2	27	0.83	1		R.RQEESYSR.M

Supplementary Table 2 GSEA analysis after sets of transcriptome analysis of PSPC1/PTK6 transfectants

**PSPC1 vs Mock**					**PSPC1/PTK6 vs Mock**					**PSPC1Y523F vs Mock**					**PSPC1-Y523F/PTK6 vs Mock**					**PSPC1-CT131 vs mock**				
**PSPC1 downstream gene dataset**					**PSPC1 downstream gene dataset**					**PSPC1 downstream gene dataset**					**PSPC1 downstream gene dataset**					**PSPC1 downstream gene dataset**				
**GSEA**	**ES**	**NES**	***P-val***	**Data origin**	**GSEA**	**ES**	**NES**	***P-val***	**Data origin**	**GSEA**	**ES**	**NES**	***P-val***	**Data origin**	**GSEA**	**ES**	**NES**	***P-val***	**Data origin**	**GSEA**	**ES**	**NES**	***P-val***	**Data origin**
**Metastasis-related signatures**					**Metastasis-related signatures**					**Metastasis-related signatures**					**Metastasis-related signatures**					**Metastasis-related signatures**				
Metastasis genes (WINNEPENNINCKX)	0.9	1.34	0.01	WINNEPENNINCKX	Metastasis genes (WINNEPENNINCKX)	-0.89	-1.21	0.018	WINNEPENNINCKX	Metastasis genes (WINNEPENNINCKX)	0.83	1.35	0.084	WINNEPENNINCKX	Metastasis genes (WINNEPENNINCKX)	0.92	1.33	0.023	WINNEPENNINCKX	Metastasis genes (WINNEPENNINCKX)	-0.83	-1.39	0.046	WINNEPENNINCKX
Metastasis genes (Rickman)	0.9	1.22	0.07	Rickman	Metastasis genes (Rickman)	-0.89	-1.32	0.027	Rickman	Metastasis genes (Rickman)	0.76	1.09	0.376	Rickman	Metastasis genes (Rickman)	0.87	1.35	0.03	Rickman	Metastasis (Richman)	-0.72	-1.31	0.05	Richman
EMT targets via CDH1 (Onder)	0.8	1.25	0.05	Onder	EMT targets via CDH1 (Onder)	-0.89	-1.36	0.013	Onder	EMT targets via CDH1 (Onder)	0.86	1.24	0.043	Onder	EMT targets via CDH1 (Onder)	0.85	1.31	0.03	Onder	EMT targets via CDH1 (Onder)	-0.65	-1.18	0.076	Onder
Metastasis genes (Tavazoie)	0.6	0.90	0.07	Tavazoie	Metastasis genes (Tavazoie)	-0.76	-1.12	0.32	Tavazoie	Metastasis genes (Tavazoie)	0.83	1.37	0.044	Tavazoie	Metastasis genes (Tavazoie)	0.97	1.40	0.005	Tavazoie	Metastasis genes (Anastassiou)	-0.86	-1.40	0.049	Anastassiou
Metastasis genes (Provenzani)	0.6	0.87	0.07	Provenzani	Metastasis genes (Provenzani)	0.65	-1.00	0.0501	Provenzani	Metastasis genes (Provenzani)	0.63	1.03	0.393	Provenzani	Metastasis genes (Provenzani)	0.91	1.35	0.022	Provenzani	Metastasis genes (Provenzani)	-0.51	-0.85	0.698	Provenzani
**Stemness-related signatures**					**Stemness-related signatures**					**Stemness-related signatures**					**Stemness-related signatures**					**Stemness-related signatures**				
Cancer stemness genes (Ramalho)	0.9	1.29	0.01	Ramalho	Cancer stemness genes (Ramalho)	-0.76	-1.12	0.321	Ramalho	Cancer stemness genes (Ramalho)	0.88	1.4	0.06	Ramalho	Cancer stemness genes (Ramalho)	0.87	1.35	0.025	Ramalho	Cancer stemness genes (Ramalho)	-0.87	-1.35	0.025	Ramalho
iPS stemness (Mikkelsen)	0.9	1.22	0.04	Mikkelsen	iPS stemness (Mikkelsen)	-0.87	-1.36	0.013	Mikkelsen	iPS stemness (Mikkelsen)	0.84	1.3	0.118	Mikkelsen	iPS stemness (Mikkelsen)	0.9	1.33	0.04	Mikkselsen	iPS stemness (Mikkelsen)	-0.77	-1.46	0.007	Mikkelsen
Stemness genes (Gaussmann)	0.8	0.98	0.64	Gaussmann	Stemness genes (Gaussmann)	-0.92	-1.34	0.01	Gaussmann	Stemness genes (Gaussmann)	0.79	1.33	0.082	Gaussmann	Stemness genes (Gaussmann)	0.84	1.27	0.089	Gaussmann	Stemness genes (Gaussmann)	-0.87	-1.34	0.098	Gaussmann
Stemness genes (Benporath)	0.8	1.29	0.04	Benporath	Stemness genes (Benporath)	-0.63	-0.99	0.5	Benporath	Stemness genes (Benporath)	0.78	1.14	0.309	Benporath	Stemness genes (Benporath)	0.65	1	0.477	Benporath	Stemness genes (Benporath)	-0.5	-0.9	0.62	Benporath
Stemness genes (Lim)	0.9	1.19	0.2	Lim	Stemness genes (Lim)	-0.6	-1.01	0.468	Lim	Stemness genes (Lim)	0.84	1.35	0.05	Lim	Stemness genes (Lim)	0.9	1.39	0.014	Lim	Stemness genes (Lim)	-0.79	-1.3	0.064	Lim
**Myc-related pathways**					**Myc-related pathways**					**Myc-related pathways**					**Myc-related pathways**					**Myc-related pathways**				
Myc targets (Schlosser)	0.9	1.34	0.01	Schlosser	Myc targets (Schlosser)	-0.8	-1.22	0.046	Schlosser	Myc targets (Schlosser)	0.81	1.32	0.09	Schlosser	Myc targets (Schlosser)	0.9	1.32	0.047	Schlosser	Myc targets (Schlosser)	-0.9	-1.3	0.004	Schlosser
Myc pathway (Lee liver cancer)	1	1.31	0.01	Lee liver cancer	Myc pathway (Lee liver cancer)	-0.86	-1.28	0.046	Lee liver cancer	Myc pathway (Lee liver cancer)	0.83	1.26	0.09	Lee liver cancer	Myc pathway (Lee liver cancer)	0.96	1.36	0.014	Lee liver cancer	Myc pathway (Lee liver cancer)	-0.91	-1.25	0.007	Lee liver cancer
Myc targets (Boylan)	0.9	1.36	0.01	Boylan	Myc-related pathways (Boylan)	-0.92	-1.35	0.012	Boylan	Myc targets (Boylan)	0.6	0.83	0.741	Boylan	Myc targets (Boylan)	0.94	1.38	0.011	Boylan	Myc-related pathways	-0.77	-1.24	0.07	Boylan
**TGF-β-related pathway**					**TGF-β-related pathway**					**TGF-β-related pathway**					**TGF-β-related pathway**					**TGF-β-related and Wnt-related pathways**				
TGF-β targets (Labbe)	0.9	1.33	0.02	Labbe	TGF-β targets (Labbe)	-0.8	-1.19	0.094	Labbe	TGF-β targets(Labbe)	0.84	1.33	0.051	Labbe	TGF-β targets(Labbe)	0.91	1.32	0.038	Labbe	TGF-β targets(Labbe)	0.84	1.33	0.051	Labbe
TGF-β pathway (GSE21670)	0.8	1.22	0.14	GSE21670	TGF-β pathway (GSE21670)	-0.88	-1.38	0.016	GSE21670	TGF-β pathway (GSE21670)	0.73	1.24	0.169	GSE21670	TGF-β pathway (GSE21670)	0.92	1.38	0.015	GSE21670	TGF-β pathway (GSE21670)	-0.54	-0.95	0.517	GSE21670
TGF-βtargets (Bhat)	0.8	1.26	0.07	Bhat	TGF-βtargets (Bhat)	-0.83	-1.23	0.156	Bhat	TGF-β targets (Bhat)	0.73	1.25	0.145	Bhat	TGF-β targets (Bhat)	0.78	1.13	0.317	Bhat	TGF-β targets (Bhat)	-0.44	-0.81	0.861	Bhat
**Wnt-CTNNB1 pathway**					**Wnt-CTNNB1 pathway**					**Wnt-CTNNB1 pathway**					**Wnt-CTNNB1 pathway**					**Wnt-CTNNB1 pathway**				
WNT signaling (Sansom)	0.9	1.27	0.04	Sansom	WNT signaling (Sansom)	-0.82	-1.35	0.013	Sansom	WNT signaling (Sansom)	0.58	0.9	0.607	Sansom	WNT signaling (Sansom)	0.78	1.2	0.141	Sansom	WNT signaling (Sansom)	-0.67	-1.28	0.041	Sansom
WNT signaling (Schaeffer)	0.8	1.47	0.05	Schaeffer	WNT signaling (Schaeffer)	-0.79	-1.31	0.034	Schaeffer	WNT signaling (Schaeffer)	0.8	1.07	0.454	Schaeffer	WNT signaling (Schaeffer)	0.76	1.17	0.183	Schaeffer	WNT signaling (Schaeffer)	-0.58	-0.98	0.5	Schaeffer
WNT targets (Smid)	0.9	1.27	0.05	Smid	WNT targets (Smid)	-0.84	-1.35	0.029	Smid	WNT targets (Smid)	0.86	1.13	0.362	Smid	WNT targets (Smid)	0.81	1.24	0.106	Smid	WNT targets (SMID)	-0.7	-1.23	0.149	Smid
WNT3A targets (Labbe)	0.6	0.88	0.1	Labbe	WNT3A targets (Labbe)	-0.8	-1.2	0.205	Labbe	WNT3A targets (Labbe)	0.79	1.37	0.045	Labbe	WNT3A targets (Labbe)	0.91	1.32	0.032	Labbe	WNT3A targets (Labbe)	-0.74	-1.24	0.036	Labbe
WNT pathways (Boyault)	0.8	1.16	0.24	Boyault	WNT pathways (Boyault)	-0.86	-1.2	0.168	Boyault	WNT pathways (Boyault)	0.95	1.31	0.047	Boyault	WNT pathways (Boyault)	0.96	1.27	0.038	Boyault	WNT pathways (Boyault)	-0.55	-0.81	0.699	Boyault
WNT targets via FGFR1(Acevedo)	0.8	1.18	0.16	Acevedo	WNT targets via FGFR1(Acevedo)	-0.72	-1.16	0.205	Acevedo	WNT targets via FGFR1(Acevedo)	0.86	1.5	0.026	Acevedo	WNT targets via FGFR1(Acevedo)	0.83	1.28	0.081	Acevedo	WNT targets via FGFR1(Acevedo)	-0.45	-0.83	0.793	Acevedo
**PTK6 downstream gene dataset**					**PTK6 downstream gene dataset**					**PTK6 downstream gene dataset**					**PTK6 downstream gene dataset**					**PTK6 downstream gene dataset**				
**GSEA**	**ES**	**NES**	***P-val***	**Data origin**	**GSEA**	**ES**	**NES**	***P-val***	**Data origin**	**GSEA**	**ES**	**NES**	***P-val***	**Data origin**	**GSEA**	**ES**	**NES**	***P-val***	**Data origin**	**GSEA**	**ES**	**NES**	***P-val***	**Data origin**
Focal adhesion	0.9	1.27	0.13	KEGG	Focal adhesion	-0.84	-1.36	0.022	KEGG	Focal adhesion	0.78	1.3	0.087	KEGG	Focal adhesion	0.87	1.35	0.027	KEGG	Focal adhesion	-0.78	-1.36	0.022	KEGG
PKA pathway(cAMP)	0.8	1.49	0	KEGG	PKA pathway(cAMP)	-0.89	-1.47	0.001	KEGG	PKA pathway(cAMP)	0.84	1.49	0.016	KEGG	PKA pathway(cAMP)	0.84	1.26	0.094	KEGG	PKA pathway(cAMP)	-0.85	-1.38	0.06	KEGG
Rac/Rho pathway	0.7	1.32	0.06	GO	Rac/Rho pathway	-0.91	-1.48	0.001	GO	Rac/Rho pathway	0.91	1.34	0.057	GO	Rac/Rho pathway	0.86	1.3	0.05	GO	Rac/Rho pathway	-0.71	-1.13	0.322	GO
Tyrosine kinase pathway	0.8	1.06	0.47	GO	Tyrosine kinase pathway	-0.93	-1.32	0.015	GO	Tyrosine kinase pathway	0.56	0.8	0.76	GO	Tyrosine kinase pathway	0.91	1.16	0.088	GO	Tyrosine kinase pathway	-0.46	-0.9	0.712	GO
GPCR pathway (GO)	1	1.35	0	GO	GPCR pathway (GO)	-0.88	-1.36	0.015	GO	GPCR pathway (GO)	0.73	1.3	0.089	GO	GPCR pathway (GO)	0.91	1.37	0.017	GO	GPCR pathway (GO)	-0.84	-1.47	0.047	GO
Receptor clustering	0.8	1.27	0.09	GO	Receptor clustering	-0.88	-1.45	0.002	GO	Receptor clustering	0.97	1.44	0.01	GO	Receptor clustering	0.92	1.36	0.021	GO	Receptor clustering (GO)	-0.9	-1.41	0.046	GO
Integrin-linked pathway	0.8	1.2	0.27	PID	Integrin-linked pathway	-1	-1.3	0.028	PID	Integrin-linked pathway	0.84	1.27	0.125	PID	Integrin-linked pathway	0.85	1.31	0.034	PID	Integrin-linked pathway	-0.85	-1.39	0.054	PID
p38/MAPK pathway	0.6	1	0.42	Phong	p38/MAPK pathway	-0.82	-1.32	0.027	Phong	p38/MAPK pathway	0.55	0.94	0.554	Phong	p38/MAPK pathway	0.61	0.94	0.608	Phong	p38/MAPK pathway	-0.84	-1.23	0.042	Phong
**HGF vs Ctrl**																								
**HGF downstream gene dataset**																								
**GSEA**	**ES**	**NES**	***P-val***	**Data origin**																				
**Metastasis-related signatures**																								
EMT targets via CDH1	0.80	1.61	0.01	Onder																				
Metastasis genes	0.9	1.54	0.02	GILDEA																				
Liver-cancer genes	0.7	1.52	0.03	HOSHIDA																				
**EMT-related signatures**																								
Metastasis genes	0.6	1.25	0.05	Hallmark																				
INVASIVENESS_SIGNATURE	0.9	1.63	0.02	ANASTASSIOU																				
TUMORIGENESIS_UP	0.8	1.45	0.05	CROMER																				
**HGF/c-MET-related pathway**																								
C-MET targets (HNA4)	0.8	1.44	0.05	SUMI																				
C-MET targets (FOXA2)	0.8	1.42	0.05	HUANG																				
C-MET targets (mTOR)	0.6	1.33	0.05	PARENT																				

Supplementary Table 3 detail IHC results of HCC patients with clinical features

Supplementary Table 3: detail IHC results of HCC patients with clinical features

							Pspc1				Pspc1 Y523				p-Brk				Wnt 3a			
CSA4							I,II		III,IV		I,II		III,IV		I,II		III,IV		I,II		III,IV	
position	age	gender	TNM	Stage	survival		L	H	L	H	L	H	L	H	L	H	L	H	L	H	L	H
1	41	M	T3N0M0	ⅢA	14	dead				1			1				1		1		1	
2	52	M	T3N0M0	ⅢA	95	alive				1			1				1		0		0	
3	56	M	T2N0M0	Ⅱ	30	dead		1				1				1				1		1
4	37	M	T4N0M1	Ⅳ	9	dead				1			1				1		1		1	
5	32	M	T3N0M0	ⅢA	3	dead				1			1				1		1		1	
6	57	M	T3N0M0	ⅢA	47	dead				1			1				1		1		1	
7	65	M	T1N0M0	Ⅰ	41	dead	1					1			1					1	1	
8	72	M	T2N0M0	Ⅱ	4	dead	1					1				1				1		1
9	58	F	T2N0M0	Ⅱ	32	dead		1				1			1					1	1	
10	52	M	T2N0M0	Ⅱ	67	alive	1					1				1				0		0
11	40	M	T2N0M0	Ⅱ	18	dead		1				1				1				1		1
12	67	M	T2N0M0	Ⅱ	18	dead	1					1			1					1	1	
13	20	M	T4N0M0	ⅢB	3	dead				1				1			1			1	1	
14	48	M	T2N0M0	Ⅱ	92	dead		1				1			1					1	1	
15	47	M	T1N0M0	Ⅰ	67	alive	1					1				1				0		0
16	69	F	T1N0M0	Ⅰ	35	dead	1					1				1				1		1
17	63	F	T1N0M0	Ⅰ	66	alive	1					1				1				0		0
18	60	F	T1N0M0	Ⅰ	47	dead	1					1				1				1		1
19	66	F	T2N0M0	Ⅱ	66	alive	1					1				1				0		0
20	64	M	T3N0M0	ⅢA	22	dead			1			1					1			1	1	
21	64	M	T1N0M0	Ⅰ	39	dead	1					1				1				1		1
22	54	M	T3N0M0	ⅢA	65	alive			1				1				1		0		0	
23	65	F	T2N0M0	Ⅱ	65	alive	1					1				1				0		0
24	41	M	T1N0M0	Ⅰ	64	alive	1					1				1				0		0
25	57	F	T2N0M0	Ⅱ	64	alive		1			1				1				0		0	
26	57	M	T3N0M0	ⅢA	64	alive				1			1					1	0			0
27	50	M	T2N0M0	Ⅱ	63	alive	1					1				1				0		0
28	43	M	T1N0M0	Ⅰ	63	alive		1			1					1			0			0
29	60	F	T4N0M0	ⅢB	63	alive				1			1				1		0		0	
30	64	M	T3N0M0	ⅢA	54	alive				1			1				1		0		0	
31	52	M	T3N0M0	ⅢA	31	dead				1			1				1		1		1	
32	54	M	T3N0M0	ⅢA	28	dead				1			1				1		1		1	
33	56	M	T2N0M0	Ⅱ	47	alive	1					1			1					0	0	
34	77	M	T2N0M0	Ⅱ	44	alive		1			1					1			0			0
35	38	M	T2N0M0	Ⅱ	28	dead	1				1				1				1		0	
36	75	M	T2N0M1	Ⅳ	3	dead				1			1				1		1		1	
37	43	M	T3N0M0	ⅢA	11	dead			1				1				1		1		1	
38	72	M	T4N1M0	ⅢC	10	dead				1			1				1		1		1	
39	60	F	T1N0M0	Ⅰ	55	alive	1					1				1				0		0
40	58	M	T2N1M0	ⅢC	1	dead		1					1					1	1			1
41	51	M	TXNXM1	Ⅳ	2	dead				1			1				1		1		1	
42	62	M	TXNXM1	Ⅳ	71	alive			1				1					1	0			0
43	61	M	TXNXM1	Ⅳ	29	dead				1			1				1		1		1	
44	60	M	TXNXM1	Ⅳ	16	dead				1			1				1		1		1	
45	47	M	TXNXM1	Ⅳ	6	dead				1			1				1		1		1	
46	75	M	T2N0M1	Ⅳ	3	dead				1			1				1		1		1	
47	43	M	TXNXM1	Ⅳ	25	alive				1			1				1		0		0	
48	72	M	T4N1M0	ⅢC	26	alive			1				1				1		0		0	
49	60	F	TXNXM1	Ⅳ	39	alive				1			1				1		0		0	
50	58	M	T2N1M0	ⅢC	1	dead				1			1				1		1		1	
51	57	M	normal																				
52	72	M	normal																				
53	58	F	normal																				
54	52	M	normal																				
55	67	M	normal																				
56	32	M	normal																				
57	63	F	normal																				
58	65	F	normal																				
59	57	F	normal																				

CSA5							Pspc1				Pspc1 Y523				p-Brk				wnt3a			
1	63	M	T2N0M0	Ⅱ	132	alive		1			1				1				0		0	
2	64	M	T3N0M0	Ⅲ	5	alive				1			1				1		0		0	
3	62	M	T2N0M0	Ⅱ	130	alive	1				1				1				0		0	
4	65	M	T3N0M0	Ⅲ	12	dead				1			1				1		1		1	
5	67	F	T2N0M0	Ⅱ	130	alive	1					1				1				0		0
6	53	M	T3N0M0	Ⅲ	126	alive			1				1				1		0		0	
7	71	M	T1N0M0	Ⅰ	80	dead	1					1				1				1		1
8	54	M	T2N0M0	Ⅱ	121	alive	1					1				1				0		0
9	69	M	T3N0M0	Ⅲ	127	alive			1				1				1		0		0	
10	57	M	T1N0M0	Ⅰ	93	dead	1					1				1				1		1
11	55	M	T1N0M0	Ⅰ	38	dead	1					1			1					1	1	
12	45	M	T1N0M0	Ⅰ	126	alive	1					1				1				0		0
13	55	M	T1N0M0	Ⅰ	36	dead	1					1				1				1		1
14	72	M	T1N0M0	Ⅰ	8	dead	1					1				1				1		1
15	66	M	T1N0M0	Ⅰ	96	alive	1					1				1				0		0
16	64	M	T1N0M0	Ⅰ	76	dead	1					1			1					1	1	
17	44	M	T2N0M0	Ⅱ	35	dead	1				1				1				1		1	
18	47	M	T2N0M0	Ⅱ	29	dead	1					1				1				1		1
19	41	F	T2N0M0	Ⅱ	53	dead	1				1					1			1			1
20	51	M	T2N0M0	Ⅱ	23	dead	1					1				1				1		1
21	64	M	T1N0M0	Ⅰ	39	dead	1					1				1				1		1
22	54	M	T3bN0M0	ⅢB	65	alive				1			1				1		0		0	
23	25	M	T2N0M0	Ⅱ	55	dead		1			1					1			1			1
24	32	M	T3N0M0	Ⅲ	31	alive				1			1				1		0			1
25	53	M	T1N0M0	Ⅰ	34	dead	1					1				1				1		1
26	62	M	T1N0M0	Ⅰ	29	alive	1					1				1				0		0
27	52	M	T2N0M0	Ⅱ	68	dead	1					1				1				1		1
28	52	M	T2N0M0	Ⅱ	35	alive		1			1				1				0		0	
29	72	M	T4N1M0	ⅣA	10	dead				1			1				1		1		1	
30	64	M	T3bN0M0	ⅢB	54	alive				1			1				1		0		0	
31	52	M	T3bN0M0	ⅢB	31	dead				1			1				1		1		1	
32	62	M	T2N0M0	Ⅱ	51	alive		1				1				1				0		0
33	56	M	T1N0M0	Ⅰ	47	alive	1					1				1				0		0
3434	7777	MM	T2N0M0T	Ⅱ Ⅱ	44	alive			1				1				1		0		0	
35	38	M	T2N0M0	Ⅱ	28	dead	1					1				1				0		1
36	75	M	T2N0M1	ⅣB	3	dead				1			1				1		1		1	
37	43	M	T3aN0M0	ⅢA	11	dead				1				1				1		1		1
38	66	M	T2N0M0	Ⅱ	54	alive	1					1				1				0		0
39	60	F	T1N0M0	Ⅰ	55	alive	1					1				1				0		0
40	58	M	T2N1M0	ⅣA	1	dead				1			1				1		1		1	
41	51	M	TxNxM1	ⅣB	2	dead				1			1				1		1		1	
42	53	M	TxNxM1	ⅣB	23	dead				1			1				1		1		1	
43	61	M	TxNxM1	ⅣB	29	dead				1			1					1	1			1
44	60	M	TxNxM1	ⅣB	16	dead				1			1				1		1		1	
45	39	M	TxNxM1	ⅣB	6	dead				1				1			1			1	1	
46	75	M	T2N0M1	ⅣB	3	dead				1				1			1			1	1	
47	43	M	TXNXM1	ⅣB	6	dead				1			1				1		1		1	
48	72	M	T4N1M0	ⅣA	10	dead				1			1				1		1		1	
49	60	F	TXNXM1	ⅣB	39	alive				1			1				1		0		0	
50	58	M	T2N1M0	ⅣA	1	dead				1			1				1		1		1	
51	63	M	Normal	Normal	.	.																	
52	64	M	Normal	Normal	.	.																	
53	62	M	Normal	Normal	.	.																	
54	65	M	Normal	Normal	.	.																	
55	53	M	Normal	Normal	.	.																	
56	71	M	Normal	Normal	.	.																	
57	69	M	Normal	Normal	.	.																	
58	55	M	Normal	Normal	.	.																	
59	52	M	Normal	Normal	.	.																	

CS4							Pspc1				Pspc1 Y523				p-Brk				Wnt 3a			
1	53	F	TxNxM1	ⅣB	100	dead				1			1				1		1		1	
2	57	M	T3aN0M0	ⅢA	8	dead				1			1				1		1		1	
3	41	M	T3aN0M0	ⅢA	14	dead			1				1				1		1		1	
4	45	M	TxNxM1	ⅣB	1	dead				1			1					1	1			1
5	54	F	TxNxM1	ⅣB	89	dead				1			1				1		1		1	
6	40	M	TxNxM1	ⅣB	6	dead				1			1				1		1		1	
7	52	M	T3aN0M0	ⅢA	95	alive				1			1				1		0		0	
8	43	M	T4NxM0	ⅢC	16	dead				1			1				1		1		1	
9	51	M	TxNxM1	ⅣB	2	dead				1			1					1	1			1
10	49	M	T4NxM0	ⅢC	89	alive				1			1				1		0		0	
11	56	M	T1N0M0	Ⅰ	30	dead	1					1				1				1		1
12	56	F	TxNxM1	ⅣB	66	dead				1			1				1		1		1	
13	40	M	T3aN1M0	ⅣA	78	alive			1				1				1		0		0	
14	37	M	T4NxM1	ⅣB	9	dead				1			1				1		1		1	
15	75	M	T2N0M0	Ⅱ	41	dead	1					1				1				1		1
16	37	M	TxNxM1	ⅣB	1	dead				1			1				1		1		1	
17	32	M	T3aN0M0	ⅢA	3	dead				1			1				1		1		1	
18	50	M	TxNxM1	ⅣB	1	dead				1			1				1		1		1	
19	57	M	T3aN0M0	ⅢA	47	dead				1			1					1	1			1
20	62	M	TxNxM1	ⅣB	71	alive				1			1				1		0		0	
21	61	M	TxNxM1	ⅣB	29	dead				1			1				1		1		1	
22	65	M	T1N0M0	Ⅰ	41	dead	1					1				1				1		1
23	64	M	T3bN0M0	ⅢB	6	dead				1			1				1		1		1	
24	65	M	T3aN0M0	ⅢA	26	dead				1				1				1		1		1
25	72	M	T2N0M0	Ⅱ	4	dead		1				1				1				1		1
26	58	F	T2N0M0	Ⅱ	32	dead	1				1					1			1			1
27	52	M	T2N0M0	Ⅱ	67	alive		1				1			1					0	0	
28	40	M	T2N0M0	Ⅱ	18	dead	1					1				1				1		1
29	53	F	T3bN0M0	ⅢB	8	dead				1			1				1		1		1	
30	67	M	T2N0M0	Ⅱ	18	dead	1					1				1				1		1
31	58	F	T3aN0M0	ⅢA	21	dead				1			1				1		1		1	
32	20	M	T4N0M0	ⅢC	3	dead			1					1				1	1			1
33	48	M	T2N0M0	Ⅱ	92	dead	1					1				1				1		1
34	47	M	T1N0M0	Ⅰ	67	alive	1					1				1				0		0
35	35	M	TxNxM1	Ⅳ	6	dead				1			1				1		1		1	
36	69	F	T1N0M0	Ⅰ	35	dead	1					1				1				1		1
37	46	M	T1N0M0	Ⅰ	49	alive	1					1				1				0		0
38	63	F	T1N0M0	Ⅰ	66	alive	1					1				1				0		0
39	60	F	T1N0M0	Ⅰ	47	dead	1					1				1				1		1
40	62	M	T1N0M0	Ⅰ	66	alive	1					1				1				0		0
41	52	M	T2N0M0	Ⅱ	13	dead		1			1				1				1		1	
42	66	F	T2N0M0	Ⅱ	66	alive		1				1				1				0		0
43	65	F	T1N0M0	Ⅰ	66	alive	1					1				1				0		0
44	64	M	T3bN0M0	ⅢB	22	dead				1			1				1		1		1	
45	59	M	T3bN0M0	ⅢB	49	dead				1			1				1		1		1	
46	64	M	T1N0M0	Ⅰ	39	dead	1					1				1				1		1
47	54	M	T3bN0M0	ⅢB	65	alive				1			1				1		0		0	
48	65	F	T1N0M0	Ⅰ	65	alive	1					1				1				0		0
49	60	M	TxNxM1	ⅣB	65	alive				1			1				1		0		0	
50	44	M	T1N0M0	Ⅰ	64	alive	1					1				1				0		0
51	41	M	T1N0M0	Ⅰ	64	alive	1					1				1				0		0
52	51	M	T1N0M0	Ⅰ	17	dead	1					1				1				1		1
53	55	M	T1N0M0	Ⅰ	71	dead	1					1				1				1		1
54	57	F	T1N0M0	Ⅰ	64	alive	1					1				1				0		0
55	57	M	T3bN0M0	ⅢB	64	alive				1			1				1		0		0	
56	43	M	T1N0M0	Ⅰ	64	alive	1					1				1				0		0
57	50	M	T1N0M0	Ⅰ	63	alive		1				1			1					0	0	
58	43	M	T1N0M0	Ⅰ	63	alive	1					1				1				0		0
59	60	F	T4N0M0	ⅢC	63	alive				1			1				1		0		0	

CS5							Pspc1				Pspc1 Y523				p-Brk				wnt3a			
1	60	F	T2N0M0	Ⅱ	109	dead		1				1				1				1		1
2	59	M	T2N0M0	Ⅱ	96	dead	1					1			1					1	1	
3	45	M	T1N0M0	Ⅰ	126	alive	1					1				1				0		0
4	45	M	TxNxM1	ⅣB	1	dead				1			1				1		1		1	
5	54	F	TxNxM1	ⅣB	89	dead				1				1				1		1		1
6	48	F	TxNxM1	ⅣB	7	dead				1			1				1		1		1	
7	66	M	T1N0M0	Ⅰ	96	alive	1					1				1				0		0
8	43	M	T4NxM0	ⅢC	16	dead				1			1				1		1		1	
9	51	M	TxNxM1	ⅣB	2	dead				1			1				1		1		1	
10	49	M	T4NxM0	ⅢC	89	alive				1			1				1		0		0	
11	64	M	T1N0M0	Ⅰ	75	dead	1					1				1				1		1
12	59	M	TxNxM1	ⅣB	128	alive				1			1				1		0		0	
13	40	M	T3aN1M0	ⅣA	77	alive				1			1				1		0		0	
14	56	M	T1N0M0	Ⅰ	55	dead	1					1				1				1		1
15	73	M	T1N0M0	Ⅰ	21	dead	1					1				1				1		1
16	48	F	TxNxM1	ⅣB	10	dead				1			1				1		1		1	
17	68	M	T2N0M0	Ⅱ	22	dead		1				1				1				1		1
18	50	M	TxNxM1	ⅣB	1	dead				1			1				1		1		1	
19	45	M	T2N0M0	Ⅱ	97	alive	1					1				1				0		0
20	52	M	TxNxM1	ⅣB	6	dead				1			1				1		1		1	
21	47	M	TxNxM1	ⅣB	33	dead			1				1				1		1		1	
22	39	F	T3aN0M0	ⅢA	5	dead				1			1				1		1		1	
23	64	M	T3bN0M0	ⅢB	6	dead				1			1				1		1		1	
24	60	M	T2N0M0	Ⅱ	21	dead		1				1				1				1		1
25	56	M	T1N0M0	Ⅰ	154	alive	1					1			1					0	0	
26	55	M	T2N0M0	Ⅱ	71	dead	1					1			1					1	1	
27	62	M	T1N0M0	Ⅰ	60	dead	1					1				1				1		1
28	52	M	T2N0M0	Ⅱ	37	alive	1					1				1				0		0
29	64	M	T2N0M0	Ⅱ	6	dead	1					1				1				1		1
30	66	M	T2N0M0	Ⅱ	173	alive	1					1				1				0		0
31	54	F	T2N0M0	Ⅱ	156	alive	1					1				1				0		0
32	56	M	T1N0M0	Ⅰ	49	dead	1					1				1				1		1
33	57	M	T2N0M0	Ⅱ	7	dead	1					1				1				1		1
34	61	M	T2N0M0	Ⅱ	122	alive	1					1				1				0		0
35	35	M	TxNxM1	Ⅳ	6	dead				1			1				1		1		1	
36	53	M	T2N0M0	Ⅱ	110	dead		1			1				1				1		1	
37	46	M	T1N0M0	Ⅰ	48	alive	1					1				1				0		0
38	65	M	T3aN0M0	ⅢA	41	dead				1			1				1		1		1	
39	56	F	T4N0M0	ⅢC	9	dead				1			1					1	1			1
40	61	M	T3aN0M0	ⅢA	10	dead				1			1				1		1		1	
41	52	M	T2N0M0	Ⅱ	13	dead	1					1				1				1		1
42	57	M	T3aN0M0	ⅢA	50	dead				1			1				1		1		1	
43	63	M	T2N0M0	Ⅱ	14	dead	1				1					1			1		1	
44	54	M	T2N0M0	Ⅱ	109	alive	1					1			1					0	0	
45	44	M	T2N0M0	Ⅱ	124	alive	1					1				1				0		0
46	64	M	T1N0M0	Ⅰ	39	dead	1					1				1				1		1
47	54	M	T3bN0M0	ⅢB	55	alive				1			1				1		0		0	
48	64	M	T3aN0M0	ⅢA	5	alive				1			1				1		0		0	
49	50	M	TxNxM1	ⅣB	17	dead				1			1				1		1		1	
50	45	M	T2N1M0	ⅣA	13	dead				1			1				1		1		1	
51	58	F	T1N0M0	Ⅰ	64	dead	1					1				1				1		1
52	64	M	T1N0M0	Ⅰ	72	alive	1					1			1					0	0	
53	55	M	T1N0M0	Ⅰ	71	dead	1					1				1				1		1
54	49	F	T1N1M0	ⅣA	6	dead				1			1				1		1		1	
55	57	F	T2N0M0	Ⅱ	121	alive	1					1			1					0	0	
56	67	M	TxNxM1	ⅣB	4	dead				1			1				1		1		1	

Supplementary Table 4 List of antibodies with their sources and experimental coditions

Protein	Assay	Antibody	Origin	Dilution	Protein	Assay	Antibody	Origin	Dilution
**PSPC1**	WB	sc-374181, Santa Cruz Biotechnology, Inc	mouse	1/1000	**Anti-phospho-PTK6 (Tyr342)**	WB	#09-144, EMD Millipore	mouse	1/1000
		sc-84576,Santa Cruz Biotechnology, Inc	rabbit	1/500	**Snail**	WB	#3879, Cell Signaling Technology, Inc.	rabbit	1/1000
	IP	sc-374181, Santa Cruz Biotechnology, Inc	mouse	1/100	**Slug**	WB	#9585, Cell Signaling Technology, Inc.	rabbit	1/1000
	IF	sc-84576, Santa Cruz Biotechnology, Inc	mouse	1/500	**Twist**	WB	25465-1-AP, proteintech	rabbit	1/1000
		SAB4200068, Sigma-Aldrich	rabbit	1/500	**Nanog**	WB	#4903, Cell Signaling Technology, Inc.	rabbit	1/1000
	IHC	sc-374181, Santa Cruz Biotechnology, Inc	mouse	1/250	**Oct4**	WB	#2840, Cell Signaling Technology, Inc.	rabbit	1/1000
**PTK6**	WB	sc-166171, Santa Cruz Biotechnology, Inc	mouse	1/1000	**Sox2**	WB	#3579, Cell Signaling Technology, Inc.	rabbit	1/1000
		18697-1-AP, Proteintech	rabbit	1/1000	**β-catenin**	WB	#8480, Cell Signaling Technology, Inc.	rabbit	1/1000
	IP	sc-166171, Santa Cruz Biotechnology, Inc	mouse	1/100	**c-myc**	WB	sc-40, Santa Cruz Biotechnology, Inc	mouse	1/1000
	IF	sc-166171, Santa Cruz Biotechnology, Inc	mouse	1/200	**Anti-Phosphotyrosine (4G10)**	WB	#05-321, EMD Millipore	mouse	1/1000
	IHC	sc-166171, Santa Cruz Biotechnology, Inc	mouse	1/100	**Vimentin**	WB	#5741, Cell Signaling Technology, Inc.	rabbit	1/1000
**N-cadherin**	WB	#13116, Cell Signaling Technology, Inc.	rabbit	1/1000	**ZEB1**	WB	#3396, Cell Signaling Technology, Inc.	rabbit	1/1000
	IF	#13116, Cell Signaling Technology, Inc.	rabbit	1/200	**p-SMAD2**	WB	#3108, Cell Signaling Technology, Inc.	rabbit	1/1000
**E-cadherin**	WB	#3195, Cell Signaling Technology, Inc.	rabbit	1/1000	**p-SMAD3**	WB	#9520, Cell Signaling Technology, Inc.	rabbit	1/1000
	IF	#3195, Cell Signaling Technology, Inc.	rabbit	1/100	**SMAD2/3**	WB	#8685, Cell Signaling Technology, Inc.	rabbit	1/1000
**Sp1**	WB	sc-420, Santa Cruz Biotechnology, Inc	mouse	1/1000	**His Tag**	WB	34670, QIAGEN	mouse	1/1000
	WB	21962-1-AP,Proteintech	rabbit	1/1000	**Anti-6X His tag**	IP	ab18184, Abcam	mouse	1/100
**HA**	WB	H3663, Sigma-Aldrich	mouse	1/1000	**β-actin**	WB	A5316, Sigma-Aldrich	mouse	1/10000
	IP	H3663, Sigma-Aldrich	mouse	1/100	**α-tubulin**	WB	A5316,Sigma-Aldrich	mouse	1/10000
**Flag**	WB	F3165, Sigma-Aldrich	mouse	1/1000	**Survivin**	WB	sc-17779, Santa Cruz Biotechnology, Inc	mouse	1/1000
	IP	F3165, Sigma-Aldrich	mouse	1/100	**Cyclin D1**	WB	sc-8396, Santa Cruz Biotechnology, Inc	mouse	1/1000
**Living Colors® EGFP**	WB	#632569, Clontech	mouse	1/1000	**goat anti-mouse IgG-HRP**	WB	sc-516102, Santa Cruz Biotechnology, Inc	goat	1/5000
	IF	#632569, Clontech	mouse	1/100	**goat anti-rabbit IgG-HRP**	WB	sc-2030, Santa Cruz Biotechnology, Inc	goat	1/5000
**γ-catenin**	WB	#2309, Cell Signaling Technology, Inc.	rabbit	1/1000	Abbreviations: IF, immunofluorescence; IHC,immunohistochemistry; IP, immunoprecipitation; mmab, mouse monoclonal antibody; WB, Western blot				
	IF	#2309, Cell Signaling Technology, Inc.	rabbit	1/100					

Supplementary Table 5 Sequences of the oligonucleotides for shRNA,real-time PCR, and site-mutagenesis.

Assay		Name		Sequence	
shRNA-52		PTK6		TACCTCTCCCATGACCACAAT	
				GCTCCGCGACTCTGATGAGAA	
shRNA-53		PTK6		CCGGAGTCGCAGAATTACATCCA	
				CCCTCGAGGGTGGATGTAATTCTGCGACTTTTTT	
Real-Time PCR	human	TGFβ1	F	CGTGGAGCTGTACCAGAAATA	
			R	TCCGGTGACATCAAAAGATAA	
		c-Myc	F	GCCACGTCTCCACACATCAG	
			R	TCTTGGCAGCAGGATAGTCCTT	
		Axin2	F	CTGGCTTTGGTGAACTGTTG	
			R	AGTTGCTCACAGCCAAGACA	
		CCND1	F	GCTGCGAAGTGGAAACCATC	
			R	CCTCCTTCTGCACACATTTGAA	
		GAPDH	F	TGTTCGACAGTCAGCCGC	
			R	GGTGTCTGAGCGATGTGGC	
		β-catenin	F	CTGAGGAGCAGCTTCAGTCC	
			R	GAGTAGCCATTGTCCACGCT	
		PTK6	F	CACAACTACCTGGCCGAGAG	
			R	GCTTCTCGCTGACCCTGAT	
		Snail	F	GGCCTAGCGAGTGGTTCTTC	
			R	GTTAGGCTTCCGATTGGGGT	
		Slug	F	TTCAACGCCTCCAAAAAGCC	
			R	GATGGGGCTGTATGCTCCTG	
		Wnt3a	F	CCCTGGAGCTAGTGTCTCCTCT	
			R	CCAATCTGTAGCCCCGCCTC	
Site-mutagenesis	human	PSPC1 Y523F	F	CCCTAATAAGCGTCGTAGATTTTAATCTAGAGGGCCCTATT C	
			R	GAATAGGCCCTCTAGATTAAAATCTACGACGCTTATTAGGG	
