# Supplementary material for: PSPC1-interchanged interactions with PTK6 and β-catenin synergize oncogenic subcellular translocations and tumor progression
Source: Nat Commun. 2019 Dec 16;10:5716. doi: 10.1038/s41467-019-13665-6 (PMC6914800; doi:10.1038/s41467-019-13665-6)
Supplement: Supplementary file 3 — Reporting Summary [file 41467_2019_13665_MOESM3_ESM.pdf]

nature research

Corresponding author(s): Yuh-Shan Jou

Last updated by author(s): 2019-11-05

Reporting Summary

Nature Research wishes to improve the reproducibility of the work that we publish. This form provides structure for consistency and transparency in reporting. For further information on Nature Research policies, see Authors & Referees and the Editorial Policy Checklist.

Statistics

For all statistical analyses, confirm that the following items are present in the figure legend, table legend, main text, or Methods section.

n/a	Confirmed	
☐	☒	The exact sample size (*n*) for each experimental group/condition, given as a discrete number and unit of measurement
☐	☒	A statement on whether measurements were taken from distinct samples or whether the same sample was measured repeatedly
☐	☒	The statistical test(s) used AND whether they are one- or two-sided<br>*Only common tests should be described solely by name; describe more complex techniques in the Methods section.*
☐	☒	A description of all covariates tested
☐	☒	A description of any assumptions or corrections, such as tests of normality and adjustment for multiple comparisons
☐	☒	A full description of the statistical parameters including central tendency (e.g. means) or other basic estimates (e.g. regression coefficient) AND variation (e.g. standard deviation) or associated estimates of uncertainty (e.g. confidence intervals)
☐	☒	For null hypothesis testing, the test statistic (e.g. *F*, *t*, *r*) with confidence intervals, effect sizes, degrees of freedom and *P* value noted<br>*Give P values as exact values whenever suitable.*
☒	☐	For Bayesian analysis, information on the choice of priors and Markov chain Monte Carlo settings
☒	☐	For hierarchical and complex designs, identification of the appropriate level for tests and full reporting of outcomes
☒	☐	Estimates of effect sizes (e.g. Cohen's *d*, Pearson's *r*), indicating how they were calculated

Our web collection on statistics for biologists contains articles on many of the points above.

Software and code

Policy information about availability of computer code

Data collection	No software was used in this study
Data analysis	no software was used in this study

For manuscripts utilizing custom algorithms or software that are central to the research but not yet described in published literature, software must be made available to editors/reviewers. We strongly encourage code deposition in a community repository (e.g. GitHub). See the Nature Research guidelines for submitting code & software for further information.

Data

Policy information about availability of data

All manuscripts must include a data availability statement. This statement should provide the following information, where applicable:

- Accession codes, unique identifiers, or web links for publicly available datasets
- A list of figures that have associated raw data
- A description of any restrictions on data availability

The gene transcription profiling data referenced during the study are available in public repositories from the Gene Expression Omnibus (GEO) database (https://www.ncbi.nlm.nih.gov/geo/).The data from the Gene Expression Omnibus (GEO) database analyzed for this study are GSE114856. All the other data supporting the findings of this study are available within the article and its Supplementary Information files. All other relevant data are available from the corresponding author upon reasonable request.

Field-specific reporting

Please select the one below that is the best fit for your research. If you are not sure, read the appropriate sections before making your selection.

☒ Life sciences ☐ Behavioural & social sciences ☐ Ecological, evolutionary & environmental sciences

For a reference copy of the document with all sections, see nature.com/documents/nr-reporting-summary-flat.pdf

Life sciences study design

All studies must disclose on these points even when the disclosure is negative.

Sample size	No statistical method was used to predetermine sample size.
Data exclusions	No samples or animals were excluded from the analysis.
Replication	all attempts at replication were sucessful.
Randomization	Randomization was not used in any experiment.
Blinding	Blinding was not used in any experiment.

Reporting for specific materials, systems and methods

We require information from authors about some types of materials, experimental systems and methods used in many studies. Here, indicate whether each material, system or method listed is relevant to your study. If you are not sure if a list item applies to your research, read the appropriate section before selecting a response.

Materials & experimental systems

n/a	Involved in the study	
☐	☒	Antibodies
☐	☒	Eukaryotic cell lines
☒	☐	Palaeontology
☐	☒	Animals and other organisms
☒	☐	Human research participants
☒	☐	Clinical data

Methods

n/a	Involved in the study	
☒	☐	ChIP-seq
☐	☒	Flow cytometry
☒	☐	MRI-based neuroimaging

Antibodies

Antibodies used	All antibodies used are listed in Supplementary Table 4.
Validation	All antibodies profiles are provided in Supplementary Table 4.

Eukaryotic cell lines

Policy information about cell lines

Cell line source(s)	Human hepatocellular carcinoma (HCC) cell lines: SK-hep1, Huh-7, SNU-387 and Mahlavu<br>Human embryonic kidney cell line: 293T and 293FT
Authentication	The authentication procedure for each cell lines were used
Mycoplasma contamination	all cell lines tested negative in mycoplasma contamination
Commonly misidentified lines<br>(See ICLAC register)	n/a

Animals and other organisms

Policy information about studies involving animals; ARRIVE guidelines recommended for reporting animal research

Laboratory animals	All laboratory mice were purchased from National Laboratory Animal Center and BioLASCO in Taipei, Taiwan and were housed and maintained under specific pathogen-free conditions.

Wild animals	n/a
Field-collected samples	n/a
Ethics oversight	All mouse experiments were conducted with approval from the Experimental Animal Committee, Academia Sinica.

Note that full information on the approval of the study protocol must also be provided in the manuscript.

Flow Cytometry

Plots

Confirm that:

☒ The axis labels state the marker and fluorochrome used (e.g. CD4-FITC).

☒ The axis scales are clearly visible. Include numbers along axes only for bottom left plot of group (a 'group' is an analysis of identical markers).

☒ All plots are contour plots with outliers or pseudocolor plots.

☒ A numerical value for number of cells or percentage (with statistics) is provided.

Methodology

Sample preparation	Cells were harvested and adjusted in fresh medium to a certain cell count per mL (1 × 106 cells/mL). Aliquots thereof were put aside for control purpose, and either verapamil (20–100µM) (Catalog number V4629), and reserpine (20–100µM) (Catalog number 83580) was added. Verapamil and reserpine are known to block several ABC drug transporters (Sigma-Aldrich).
Instrument	LSR II SORP or Fortessa (BD)
Software	data analysed using FlowJo software
Cell population abundance	Mentioned in the Methods section
Gating strategy	Mentioned in the Methods section

☒ Tick this box to confirm that a figure exemplifying the gating strategy is provided in the Supplementary Information.
